# Digital technology adoption scale in the blended learning context in higher education: Development, validation and testing of a specific tool

**DOI:** 10.1371/journal.pone.0235957

**Published:** 2020-07-10

**Authors:** Iuliana Mihaela Lazar, Georgeta Panisoara, Ion Ovidiu Panisoara

**Affiliations:** 1 Department of Teacher Training, Faculty of Psychology and Educational Sciences, University of Bucharest, Bucharest, Romania; 2 Psychology Department, Faculty of Psychology and Educational Sciences, University of Bucharest, Bucharest, Romania; Univerza v Mariboru, SLOVENIA

## Abstract

The main aim of the present study was to develop, validate and test an extended Technology Acceptance Model (TAM) that contributes to the overall understanding of students' intention to use digital tools in a blended learning context of higher education. The external bidimensional factor of familiarity with digital tools, which is not usually explained by the TAM, was included, and evaluated. Following a four-stage scale development technique, a seven-dimensional 25-item survey was developed, which includes two external correlated variables: familiarity with high-tech digital tools and familiarity with traditional digital tools, two mediator variables—computer anxiety, and perceived barriers, and three response variables, perceived usefulness, perceived ease of use and intention to use. The initial version of the survey was administered on 250 undergraduate students. Next, for another sample of 206 students, latent dimensions of the survey were tested using exploratory factor analysis. The structure of the survey was validated in two other subsequent stages with one sample of 262 responses of undergraduates and one of 310 responses of master's students from two different universities. All students who agreed to participate in research attended blended learning. The validity, reliability and invariance of the instrument were established by psychometric analyses. Collected data indicated that the survey has an adequate multifactorial structure that is reliable and invariant across degree levels. The scale is recommended for use in higher education studies targeting the promotion of blended learning and reduction of negative attitudes of learners toward digital instruments, supporting university professors to select their own efficient way to teach.

## Introduction

The use of digital technology in education already has several decades of history. Despite this, from the pedagogical point of view, many questions are yet to be answered. The response of the students when these technologies are used in the learning process is influenced by many factors. These constructs undergo a continuous transformation due to the change of the actors of the educational process. Rapid changes can be observed related to the digital competencies of users and their attitude toward technology.

One of the first accepted models used to evaluate the adoption of digital technology was presented by Davis in 1989 [1]. The model was applied in education, where computers and digital technology started to be used. The initial version of the Technology Acceptance Model (TAM) was continuously developed and adapted depending on the technological and social evolutions, but also to offer reliable results for different activities. First models adapted for education were used to evaluate the introduction of computers in the learning process [2–5]. The development of the Internet and the burst of e-learning systems imposed some adjustments regarding the theoretical framework and the instruments used to study the digital technology adoption [6–8].

The introduction of interactive textbooks [9] and the limitations of the e-learning system led to the emergence of a blended learning system, in which the traditional face-to-face learning system is combined with e-learning specific instruments [10]. The "Transnational Needs Analysis Report" coordinated by the Centre for the Advancement of Research & Development in Educational Technology, Cyprus mentions blended learning as a" *novel practice that is mainly used in higher educational institutions*" [11].Even if according to Law et al. [12]" *the blended learning approach has a significantly positive influence on learning performance*, *students' satisfaction was found to be higher in blended courses than the traditional classroom teaching*, *and blended learning enhanced students' learning process*", only few studies on acceptance of blended digital technologies in higher education were published [13, 14]. Moreover, very few studies have been conducted on students in universities [15, 16] as end-users of digital tools in a blended learning context. Until now, researchers of digital tools have focused especially on potential benefits and challenges for higher education.

The demand for blended training is increasing along with the demand to adapt learning to individual needs in different social, economic and cultural contexts [17]. In the literature dedicated to education, researchers have not yet developed a theoretical construct to identify students' behavior towards the simultaneous use of face-to-face and e-learning technologies. This construct is necessary because at this moment the empirical research is still dominated by individual technologies such as e-learning, m-learning, or face to face learning, which do not reflect the latest educational strategies that involve the use of mixed resources [18].

The efficient use of mixed educational resources for teaching [19, 20] represents a real challenge for teachers. But it is necessary to identify the most suitable digital resources [21] to maximize the amount of acquired knowledge. Also, it is particularly useful to understand the effect of external and mediating variables that act on the intent of using digital resources in the process of learning among students in higher education.

Depending on the type of digital technology used, on a scale from total disapproval to total approval, undergraduates and graduates have different opinions. Thus, it is essential to recognize the features that encourage all students to use digital tools to create an effective learning environment [22]. To do this, researchers have used a number of different theories and models on acceptance of information technologies (IT) such as Theory of Reasoned Action (TRA), Technology Acceptance Model (TAM), Theory of Planned Behavior (TPB), Decomposed Theory of Planned Behavior (DTPB), Uses and Gratifications Theory (UGT), Unified Theory of Acceptance and Use of Technology (UTAUT), Innovation Diffusion Theory (IDT), Expectation-Confirmation Theory (ECT) and others [23–25].

The TAM model, as an expression of the interest in knowing how a user is referring to technology innovation, has been periodically developed and adapted to different needs. The amount of research that developed, validated and confirmed the TAM has expanded rapidly over the last decades [26–35]. Universities are reacting in different ways in blended environments. Learners expectations are adjusted differently in various educational contexts. Quick transition from face-to-face learning to distance learning, like in pandemic times has been found problematic and challenging [18]. A very good planning and fast adoption of teaching and learning strategies to the contexts must be followed. This means that permanent studies are needed to identify which external factors can significantly influence the adoption of technologies for mixed learning contexts.

Usually, each study has developed its own research methodology to measure user acceptance. However, these investigations did not deepen the issue of blended learning tools in higher education. Moreover, the influence of familiarity with blended learning tools mediated by risk factors is not described. The development of special scales for testing the adoption of mixed digital tools is desired because they offer the possibility of development for comparative studies between traditional and high-tech tools for specific populations (e.g., students in social science fundamental domain) with general intents (e.g., the measurement of intention to use tools in a blended learning context of higher education). In this context, the main aim of the study was to present the development, structural validation and path testing of a scale projected to measure the acceptance of digital tools in a blended learning context by university students, incorporating all the five criteria to guarantee product quality: simplicity, good reliability, good construct validity, invariance and reproducibility [36]. The scale constructed based on the basic TAM, was named the Blended Learning Scale (BLS), and explores the adoption of high-tech and traditional digital tools mediated by the perceived barriers and computer anxiety among students in higher education.

As the main novelty, the proposed model uses an original pair of external variables, the familiarity with traditional instruments used in face-to-face education, and the familiarity with advanced digital instruments used in education. Another point of originality is the use of computer anxiety and perceived barriers as moderating factors.

The research identified, measured, and evaluated the existing correlations among the components of external variables, mediator variables and the TAM constituents relative to digital technology usage behavior in a blended learning context.

## Theoretical background

There are three main issues to be discussed related to the proposed subject: the model used to build the scale, the specific characteristics of the investigated learning process, and the variables used to link the model to the process. A literature study was conducted to identify the best model to use and the main traits of the blended learning context.

### The model

The first challenge was to choose a model as starting point to construct the research instrument. The number of theories and models on acceptance of information technologies used by researchers is very large, and each model presents a lot of variants and extensions. Analyzing the literature, one conclusion becomes clear: the theoretical model must be tailored as a function of the particularities of the investigated subject. A non-systematic bibliographic study was conducted to identify the best base model to start with. Several main electronic databases have been accessed for search, including ScienceDirect, Springer, ProQuest, and Google Scholar, using "adoption", "tools", "students", "higher education" and "digital" as keywords. To guarantee the reliability of studies for data analysis, the following criteria [37] were used: a) the studies had to have explored the adoption of digital tools, b) the target group had to be composed by university students, c) the trials had to be published in prestigious journals, and d) the scale of adoption had to be used later in the context of other research, which confirms the validity of the scales [36].

On the whole, throughout a 6 years span (2013–2018), 34 studies on university students related to Information and Communications Technologies (ICT) tools acceptance have been identified. The table containing the information extracted from each study can be seen in the S1 Appendix. For each reference there have been collected data on the analyzed context, geographic area of the study, the model used, external, mediating and response variables, size and composition of the target group. Special attention was also given to the model used in the studies and to the external variables that may influence the adoption of ICT tools.

The main finding was that most of these studies (21 of 35, 62%) used different variants of the TAM to analyze the proposed subject. Assuming this, an Extended TAM for measuring levels of learners' acceptance of digital tools for higher education in a blended learning context was selected for this study. This model can be largely useful and appropriate to many digital technologies used in blended learning and should be constructed based on the most appropriate external and moderating factors identified during research.

On average, scales for measuring the adoption of IT tools are designed to capture people's behaviors, attitudes, or internal emotional conditions [38]. The use of the TAM can identify to what extent students accept and use technology and the manner and time they choose to use it. Also, the factors that affect university students' resistance to using technology [39] can be identified.

In principle, TAM assumes that the *intention to use a tool*, as the final output variable of the model, is influenced by two main factors: *perceived ease of use* and *perceived usefulness* [40]. Thus, in the Extended TAM, the three main factors, *perceived ease of use*, *perceived usefulness*, and *intention to use* were considered as *response variables*, being the result of the action of several *external variables*, describing cognitive instrumental processes, under the influence of some *mediating variables*, coming from the social influence [41]. To establish the best external and mediating variables for our model, some important aspects of the blended learning activity were considered.

### The subject

Blended learning was interpreted as a third generation in the evolution of higher education, the first one being the traditional, face-to-face education, and the second the e-learning education [42, 43], but it is more and more a reality for all levels of education. Blended learning tools are resources that mediate regular face-to-face education with web-based online learning. A large part of the content designed for blended learning is delivered online (between 30% and 80%) [44]. In this logic, the blended educational strategy can be appreciated as the current method to teach and learn.

The blended learning process is a consequence of the impetuous development of digital technologies and digital educational tools (DETs), in line with the evolution of society. In Blake and his collaborators`point of view, the term digital educational tool (DET) can include "*electronic books*, *online journals*, *movies*, *reference texts such as dictionaries as well as audio or image files; it is used to cover material created digitally or by scanning analogue resources*" [36]. Later, Morais and collaborators found that it is not easy to express a consensual description of DET because of the complexity of perception and the multi-dimension that it contains [36]. What we have stated with certainty is that DET is a resource in an electronic form that can be adapted to the needs of learning or other educational purposes.

These DETs can be lesson plans, lectures, books, textbooks, homework, portfolios, university resources, software, quizzes, tests, resources, audio, and video clips, and more, all available in digital format, with or without free access. As Watling points out, social media like Twitter, YouTube, and Facebook also become DETs [45].

In a similar approach, Blas et all [37] highlight that over the last decades, a great number of repositories of educational resources have prospered on the Internet. At the same time, considerable adjustments were perceived in education methods and original types of resources become available, all of them constructed on digital knowledge. DETs have been classified into various hierarchies like "*educational software*, *educational platforms*, *portals of contents*, *learning tutorials*, *electronic files and thematic resources directories*, *guidelines*" [46, 47], but the most common difference between digital tools consists in the way they are designed and they can be reproduced [46].

In this paper, the term '*digital learning tool*' is used to describe all digital resources potentially beneficial in the strategy of blended learning, which include:

- *High-tech digital learning tools*, including software that supports student learning (e.g., interactive boards, scientific software, apps, digital coursewares, digital textbooks, mobile devices (smartphones and /or tablets), etc.), and
- *Traditional digital learning tools* (e.g., digital video support, video overhead projectors, interactive materials, digital collections containing interactive resources and reference texts such as lecture notes or dictionaries, etc.).

### The variables

The analyzed studies on the acceptance of digital tools among university students presented in the S1 Appendix use an important variety of output and mediating variables in a stronger or weaker relation with the external variables, like Self-Efficacy, Subjective Norm, Enjoyment, Computer Anxiety and Experience, Learning Content Quality, Interactivity, Content Design Quality, User-Interface Design, Attitude Towards Use, Perceived Technical Characteristics of Digital Tools, Support Efficacy, Performance Expectancy, Effort Expectancy, Social Influence, and others. For the present research, the external variables, describing cognitive instrumental processes, must be related to both classic and high-tech instruments used in a blended learning environment, and the social factors acting as mediating variables must be related to the anxiety and perceived barriers in the use of digital resources.

#### External variables

Most of the students pertaining to the higher education system has obvious skills regarding the use of different DETs. In a blended learning environment, the use of digital instruments is a necessity. The classic face-to-face learning also uses more or less digital instruments. The question is which factors influence the perceived usefulness and ease to use of these different instruments. One way is to identify as many external variables as possible. Still, a scale built on such an idea is challenging to be analyzed from a statistical point of view. Another way, chosen as a solution for this research, was to find the concepts that summarize the most part of the identified variables.

One of the important concepts describing a cognitive instrumental process related to the use of digital instruments in blended learning is *familiarity*, even if "*the concept of familiarity is not well researched in the technology adoption literature*", as stated Abu-Shanab [48].

Starting from these statements, this research proposed as external variables *Familiarity with classical digital tools* (Traditional) and *Familiarity with high-tech digital tools* (Modern). While many studies approach the integration of online digital learning into universities, fewer have regarded the simultaneous integration of both traditional and high-tech digital learning technologies. This might be challenging at a time when universities implement more and more the blended learning system, using traditional and high-tech digital learning technologies in both face-to-face and online learning.

#### Mediating variables

The challenge of reaching the highest level of learning in the shortest time possible and with the lowest effort seems to be a strong motive for learners to want to use digital technologies. The success of an advanced instrument is not just understood in rapports of purpose and practice but also takes into account whether the tool is able to satisfy or not the requirements of users. Therefore, the significance of exploring the feelings of users cannot be neglected.

One factor found as significant in many studies on the adoption of digital tools and adopted for this study is *computer anxiety*. The research on computer anxiety is a concept that has been largely studied after the beginning of the Digital Revolution in the 1980s and is still applicable nowadays [37, 49, 50]. A comprehensive review of the primary concepts in computer anxiety was published by Powell in 2013 [50], who proposed several variables as predictors of computer anxiety.

The concept of *computer anxiety* suggests that it is shaped as a reply to perceived threats from a technology that may be too hard to use and that the performance benefits of usage are outweighed by their effort. That will significantly affect the user's behavior, experiencing less pleasure when thinking about using digital tools [51] and increasing the concern related to its use [52].

Another influencing factor adopted for this research, less presented in other models, was the *perceived barriers*. Studying the barriers in using digital tools can offer proper ways to understand the adoption of digital teaching-learning technologies. This understanding may offer support in regular educational activities [53]. One prominent challenge comprises constraints and limitations imposed by the financial aspects, respectively, the costs of different digital tools. Other significant challenges about digital accessibility include the quality of digital tools and time effectiveness for learning to use them. Finally, overall dominant challenges are related to the lack of instructions and competences to use specific digital tools.

#### Internal variables

Basic TAM uses two response variables, *Perceived Usefulness (Usefulness)* and *Perceived Ease of Use (Ease)*, both influencing the final exit variable *Behavioral Intention to Use (Intention)* [1]. The two response variables are not independent, *Perceived Usefulness* usually influencing *Perceived Ease of Use*, but not vice versa.

The perception of ease of use and usefulness of digital educational technologies are consequences of the interaction between users and technology during the educational process.

The study of end-user’s behavior in higher education requires an integrated approach that needs to consider different kinds of learners' perceptions and used tools.

## Methods

The development and validation of a specific metric involved two different aspects from the methodology point of view. In the first part of the present research, how the research instrument was developed was presented. Next, the relevant information of data collection was described, and quantitative data analysis were discussed.

### Research model

The present research used an extension of the TAM to investigate the use of digital tools as learning resources in a blended learning context. The purpose of this extension of the basic TAM was to identify and explain as accurately and correctly as possible the factors that influence the user decision regarding digital tools in a blended higher education context.

Constructed on previous researches, the present research has extended the variables used to examine learners' weaknesses within the theory of acceptance and use of technology. Consequently, one investigation model was developed to interpret the structural effects of familiarity with high-tech and traditional learning tools, which are external dimensions, on the intention to use DETs, ease of use, and usefulness of DETs, which are internal dimensions mediated by computer anxiety and risk perceptions. Accordingly, the secondary aim of this research was to examine which external variables facilitate the adoption of DETs among university students.

Briefly, the research model has two original external variables, two less used mediating variables, and three internal variables, *Perceived Usefulness*, *Perceived Ease of Use*, and *Behavioral Intention to Use*. The concept of attitude towards use was excluded due to its reduced effect, in order to simplify the model and diminish the number of items on the survey [54].

### Instrument development

A multi-stage method was used to develop the research instrument that measures digital technology adoption among university students in a blended learning context. The type of research tool used in this study was the questionnaire. The development of the instrument was realized using four different samples, one for each development stage, organized into two campaigns, during January-June 2017 and January-June 2018. Each questionnaire was administered paper-and-pencil and online and the participation of all respondents was voluntary.

The procedure of instrument development involved four steps. The instrument was constructed based on psychometric analysis using three samples of undergraduates and one sample of graduate students as participants described in the previous subparagraph. The first three samples were used for the development and internal validation of the instrument and the last one was used for the external validation of the scale. The suggested psychometric procedure consisting of several separate stages found in the literature [36, 55] was used to develop a reliable and valid version of the research instrument namely the Blended Learning Scale (BLS). Accordingly, four separate stages were used for the instrument development: stage I (*preliminary stage—writing the proposed items according to the theoretical context and testing the adequate content validity and reliability using Items Analyses*), stage II (*estimation of latent structure through Exploratory Factor Analyses*), stage III (*testing the validity of the measurement scale using Confirmatory Factor Analyses (CFA)*, *and explored the links between factors using Structural Equation Modelling (SEM) and Path Analyses*), stage IV (*testing the replication generalizability of the measurement scale using the same multivariate analyses (CFA*, *SEM and Path Analyses*). The activities carried out, the methods used, the samples used for questionnaire, and the results obtained are summarized in S2 Appendix.

### Samples

Data were collected in two universities, the University of Bucharest (UB), and the Vasile Alecsandri University of Bacau (UVABc), both from Romania. The main criteria for asking undergraduates and graduates to join the research were to be actively engaged in blended learning courses. All participants decided to participate voluntarily in the research and, before starting, they were informed about the aims and research methods accordingly with the ethical guidelines [56]. Four hundred twelve undergraduate students of engineering studies from the UVABc were invited to participate during January-June 2017 in a pilot test to develop the Initial BLS scale model. Then, exploratory factor analysis (EFA) was conducted on 352 undergraduate students from Education Science studies, UB to identify the dimensions of the Initial BLS scale model. During January-June 2018 the constructs of the BLS scale were validated by confirmatory factor analysis (CFA) using a separate sample of 414 undergraduate students from Psychology studies, UB). Finally, 511 graduate students from Education Science studies and Environmental Science studies, UVABc were recruited to participate in the last stage of research. A total of 250 valid respondents were retained for Stage I, 206 valid respondents were retained for Stage II, 262 valid respondents were retained for Stage III and 310 valid respondents were retained for Stage IV by eliminating those who had given invalid answers [57], resulting in a satisfactory answer percentage of approximately 63.5%.

### Measures

#### Familiarity with high tech and traditional digital tools

In this study, the original TAM model was modified by including two correlated external variables, based on the literature search and the particularities of blended learning. The digital instruments were divided into two categories, *traditional tools*, DETs usually used in the learning process, and *high-tech tools*, digital instruments less used in the classrooms in the last years.

High-tech tools can also be defined as *adaptive* tools. Based on a literature review and the high-tech instrument used in the universities participating in the research, several instruments were chosen to evaluate this variable. Examples of such high-tech instruments are the new generation of interactive learning tools (interactive board and e-textbooks), cloud-based service tools (Google Drive, Docs and Earth), scientific software (IBM SPSS® software), social media apps and sites, or the new generation of computer and mobile devices (smartphones and tablets).

Traditional tools can be defined as the digital tools used for face-to-face learning activities. The operational definition of this factor consists of classical digital tools used as educational resources that are proper for digital learning. In the case of traditional tools, the instruments chosen to investigate the familiarity were some of the main resources used in daily activities, like audio and video equipment, television, overhead projectors, digital face-to-face exercises and games, or laptops and computers used to access digital course materials.

#### Barriers and anxiety

This version of the TAM model proposes two mediating variables related to social factors influencing the use of digital resources, both in a negative way.

*Barriers* referred to a set of economic, social and educational factors that can obstruct a specified behavior. Some barriers analyzed in this research (such as financial risks, quality risks, time effectiveness and knowledge risks) will influence the users' intention to adopt DETs.

This factor was evaluated using items such as *Cost of access limits*, *Poor quality of digital educational tools*, *Lack of awareness of invested time for use*, *Lack of awareness of copyright issues and Lack of digital competence*.

*Anxiety* referred to a possible emotional outcome experienced at a lower or higher level by the persons using DETs and had three dimensions: nervousness, unpleasant feelings, and uncomfortable feelings. These negative feelings were translated into three items, adopted from previous research [58]: "*Working with digital tools makes me nervous*", "*Digital tools give me an unpleasant feeling*", and "*Digital tools make me feel uncomfortable*".

#### Usefulness, ease, and intention

Apart from providing their socio-demographic data (age, gender, university, degree level and field of study), participants answered to 17 statements on respondent variables (i.e., Perceived usefulness (five items), Perceived ease of use (nine items), and Behavioral Intention to use (three items)). The items were adapted from related literature or self-developed (S3 Appendix). The measures of digital tools adoption focus on easily to acquires of useful skills for DETs usage, to usefulness of digital tools usage such as enhancing of self-education, improving user’s knowledge exchange or intention to use different digital tools, only after the documentation about the DETs usage.

### Questionnaire

According to Black and Champion (1976) [59], the first draft of the questionnaire was revised based on the opinions of internal and external evaluators. This research adopted or self-developed items to measure familiarity with high-tech and traditional digital tools [60, 61], items to measure perceived barriers [62], items to measure computer anxiety [37, 51, 63], items to measure perceived usefulness [27], items to measure perceived ease of use [1, 64], and items to measure behavioral intention to use [58]. Eight external experts in the field of education from South Africa, Slovenia, Cyprus, and Spain and four internal experts from the UVABc and UB were asked for validity content check of survey. The request was to rate each proposed item on a 10-point grading scale for applicability, objectivity, and relevance, and with" yes" or" not" how difficult it is to understand the item. They also were invited to propose strengths, weaknesses, and observations. All internal and external evaluators were not involved in writing the items, but only in their review. Based on their reviews, each item has been evaluated. First, the average of scores awarded for the three criteria was calculated for each evaluator and each item. In the second step, all items with the average score below 5 calculate for two or more evaluators and the items with the answer "not" for the fourth question were eliminated. Based on the experts' opinions some items were rewritten. The structure of the questionnaire established at the end of this step containing 36 items () was sent for a second revision. All the items were measured using a five-point Likert type scale from 1 = Completely Disagree to 5 = Completely Agree. The experts gave positive feedback on amendments and provided no additional observations. Briefly, the 36-item research questionnaire (S3 Appendix) tool was used to conduct the surveys to understand the students’ perceptions and intentions regarding the usage of digital tools in blended learning context.

### Data analysis methods

Data analysis was made using IBM SPSS with license +SW version 20.0 and IBM® SPSS®

Amos free version 26. Different analysis methods were used in different stages of scale development, validation, and test.

#### Nonparametric test

The purpose for nonparametric test usage was to check differences between two or more than two groups comparing population mean ranks. For this, trends in the total scores associated with all testing items were examined using the Mann–Whitney test and Jonckheere-Terpstra test [25]. So, the homogeneity of the subjects was evaluated using nonparametric tests for the first and last stage, where the test groups included university students from different programs of study.

#### Item analyses

In the first stage of scale development, separate item analyses were performed for inspection of the distributions of answers for each item and the relation of each item with the others. In this situation, the item-total correlation, and the items whose item-total correlation was below the cut-off criterion of 0.20 were excluded from the questionnaire as described by Türel [65].

The total content validity index (CVI) calculated for the pilot study was 0.86, bigger than the threshold value of 0.80 proposed by Chang [66].

#### Exploratory Factor Analysis (EFA)

Exploratory Factor Analysis (EFA) [66, 67] was used to test the combination of items, identifying the best model that explains the intention to use blended learning tools [27]. The EFA was conducted using the selection criteria of an eigenvalue greater than 1.0 and the factor loadings for each item greater than 0.6 [68, 69]. The Principal Component Factor (PCA) method, followed by the Promax rotation, which is the most used form in the literature, gave a better solution for the EFA. Exploratory factor analysis led to the identification of seven factors based on the Kaiser's criterion [57] resulted after oblique rotation by the Promax method extraction method.

The sampling suitability of the study was confirmed by the Kaiser-Meyer-Olkin (KMO) measure and Bartlett's test of sphericity [70]. Finally, the internal consistency reliability (the significance level α corresponds to 0.05) and the KMO measure of sampling were calculated for the entire questionnaire as well as for each latent factor.

#### Confirmatory Factor Analysis (CFA)

The Confirmatory Factor Analysis (CFA) based on the maximum likelihood estimation [71] was conducted with AMOS 26.0 free version statistic package to test the best model to represent the experimental data. Also, the reliability and validity of the dimensions were estimated using CFA [72]. Multiple criteria to evaluate the goodness-of-fit of data to the measurement model, as suggested in the literature, were used [73]. Several indices (Chi-square/degree of freedom (χ^2^/df), Tucker-Lewis index (TLI), Comparative Fit Index (CFI), standardized root mean square residual (SRMR), and the root mean square error of approximation (RMSEA)) were examined to evaluate the measurement model that that best fits the data [74].

Composite reliability (CR), Average Variance Extracted (AVE) and Maximum Shared Variance (MSV) were the indicators used to test the construct validity. Indicators were calculated using formulas presented by Lee [75]. Maximal Reliability (MaxR(H)) a measure of maximum reliability [72] as an alternative reliability index to coefficient alpha was also measured [76]. The accepted values were 0.7 or higher for the CR and MaxR(H) and), and 0.5 for AVE [77]. The magnitude of the links between latent factors determined the discriminant validity of the variables [75]. According to Fornell and Larcker [78], discriminant validity is fulfilled when "*each concept's AVE is greater than the squared correlation of the construct with any other construct*". Discriminant validity at item level is additional proved when MSV < AVE [79]. Therefore, it was explored whether the square root of the AVE of each variable was higher than the inter-factor correlations to test the discriminant validity [77].

#### Path analysis

Testing the links between the dimensions of the BLS final scale was performed using a structural equation modelling technique. This analysis offers information on the relation between latent factors, but also on the nature of the relationship between the predictors and latent factors. The proposal for a measurement model and its confirmation in the framework of SEM structural equation modelling generates an increase in the accuracy of the factor estimation and the validity of the study conclusions [80]. In this research, the path analysis was used to identify factors influencing dependent variables (perceived usefulness, perceived ease of use and behavioral intention to use) [81]. Therefore, the measures corresponding to Stages III and IV were subsequently subjected to a path analytic model to validate the hypothesized relationships between factors [82]. Accordingly, the method allowed us to make comparisons between independent groups (i.e., undergraduate versus master's students), to quantify and compare the effects of external variables on each of the internal variables moderated by mediating variables [83].

### Research hypotheses

The research was mainly interested in developing a valid and reliable instrument to explore the key factors influencing blended learning adoption in higher education, and to test the relationship between the tool constructs. To complete the research aim, fifteen specific research hypotheses were expressed (Table 1).

**Table 1:** Indicates the hypothesized path, corresponding code and details.

Hypothesized path and corresponding code		Details
Traditional→Barriers	H1	The familiarity with traditional digital tools has a positive impact on the learner's barriers perception
Modern→Ease	H2	The familiarity with high-tech digital tools has a positive impact on the perceived ease of use
Anxiety→Ease	H3	Computer anxiety has a negative impact on the perceived ease of use
Anxiety→Barriers	H4	Computer anxiety has a positive impact on the learner's barriers perception
Anxiety→ Usefulness	H5	Computer anxiety has a negative impact on the perceived usefulness
Barriers→Usefulness	H6	The learner's barriers perception has a positive impact on the perceived usefulness
Ease→Usefulness	H7	The perceived ease of use has a positive impact on the perceived usefulness
Traditional→Usefulness	H8	The familiarity with traditional digital tools has a positive impact on the perceived usefulness
Ease→ Intention	H9	The perceived ease of use has a positive impact on the behavioral Intention to use
Usefulness→ Intention	H10	The perceived usefulness has a positive impact on the behavioral Intention to use
Traditional→ Intention	H11	The familiarity with traditional digital tools has a positive impact on the behavioral Intention to use
Modern→ Usefulness	H12	The familiarity with high-tech digital tools has a positive impact on the perceived usefulness
Modern→ Intention	H13	The familiarity with high-tech digital tools has a positive impact on the behavioral Intention to use
Anxiety→ Intention	H14	Computer anxiety has a negative impact on the behavioral Intention to use
Barriers→ Intention	H15	The learner's barriers perception has a positive impact on the behavioral Intention to use

### Ethics, consent, and approvals

The subject of the study and the organization of the research in relation to the participants to surveys were approved by the Commission of Ethics and Academic Professional Deontology from the UB. The tool used to ask for the responses was Google Forms (https://docs.google.com/forms). All participants voluntarily agreed to participate in the research and no personal data were collected because the survey answers were totally anonymous. Participants were informed during the dissemination of the surveys of the aims of the study and were asked to take part voluntarily, having the option to withdraw from the study at any time [56]. For each survey, only aggregate information was used and published.

## Results

### Participant characteristics

The first sample, corresponding to Stage 1, involved undergraduate students in three branches of Sciences: Environmental Science (32.8%), Chemical Science (22.8%) and Computer Science (44.4%) at the Vasile Alecsandri University of Bacau, with an average age of 24.03 years old, female (40% of total) and male (60% of total). The second sample, corresponding to Stage 2, involved undergraduate students in Educational Sciences at the Bucharest University, with an average age of 25.04 years old, female (98.5% of total), and male (1.5% of total). The third sample, corresponding to Stage 3, involved undergraduate students in Psychology at the Bucharest University, with an average age of 22.95 years old, female (89.9% of total), and male (10.1% of total). The fourth sample, corresponding to Stage 4, involved master's students in Educational Sciences and Environmental Science at the Vasile Alecsandri University of Bacau, with an average age of 30.53 years old, female (82.5% of total) and male (17.5% of total). Thus, the present research had a total sample size of 1037 subjects, which can be viewed as adequate [65]. The datasets are provided in S4 Appendix. The homogeneity of the group of participants to surveys was assured by the selection of target groups among students from the same field of studies and the same degree level. The homogeneity of the samples composed by different groups was evaluated, and the results are presented in the next paragraph.

### The homogeneity of the participants

The homogeneity of the participants on research was evaluated in the first and last stage, where the samples contained students from different study fields. Trends in the total scores associated with all testing items were examined using the Jonckheere-Terpstra test. The results showed no significant differences for participants in Stage 1 between the three fields of Sciences: Environmental Science, Chemical Science, and Computer Science. The homogeneity of the sample of Stage I was confirmed (the total scores of 36 items: number of levels = 3, 𝑁 = 250, Mean J-T statistic = 10051.500, Monte Carlo Sig. (2-tailed) = 0.702). Similar to Stage I, the results showed no significant differences for participants in Stage 4 between the two fields of Sciences, Educational Science and Environmental Science (the total scores of 25 items: 𝑁 = 310, Wilcoxon W = 24343, Z = -1.472, Asymp. Sig. (2-tailed) = 0.141).

### Results of the pilot test and item analyses

At the beginning of the development process of the BLS university student questionnaire, studies regarding the adoption of digital tools used in high school backgrounds were examined in order to generate an initial pool of items based on a comprehensive review of research papers published in technology adoption and related subjects. Items identified via one-on-one debates and previous studies were assembled to generate the initial version of the item pool (121 items). Based on ten experts' contribution, these items were revised to fit the features of the BLS scale. They contracted the size of the initial pool to 36 items (10-item to measure the familiarity with high-tech and traditional digital tools, 5-item to measure the perceived barriers, 4-item to measure the computer anxiety, 5-item to measure the perceived usefulness, 9-item to measure the perceived ease of use, and 3-item to measure the behavioral intention to use). The total content validity index (CVI) calculated for the pilot study was 0.86, higher than the threshold value of 0.80 proposed by Chang. Then, the 36-items questionnaire (S3 Appendix) was pre-tested using the first sample. The results of the analysis of the questionnaire regarding mean, standard deviation, skewness, kurtosis, corrected item-total correlation for each item and Cronbach's alfa coefficient of the scores of each subscale from Stage I (N = 250) are presented in the S5 Appendix. Another four items were eliminated because of the low value of the corrected item-total correlation (below 0.20) [65] and also based on the opinion of the external evaluators, resulting in a final model at the end of Stage I with 32 items.

### Initial BLS scale dimensionality

In the next step, the Exploratory Analysis was constructed on 32-itemBLS scale and 206 participants. After 8 iterations, seven extracted factors described 62.25% of the change in the items. The Bartlett's Test of Sphericity (BTS) presented an approximately Chi-Square value of 4592.463 (p < .001), which meant that the correlation matrix of data for factor analysis was suitable.

From the evaluated BLS scale, seven items have been removed due to low factor loadings and cross-loading concern [68]. Finally, 25 items in seven factors have been kept, as shown in Table 2.

**Table 2:** The items of the Initial BLS Scale model and the related references.

Category of factors	Factors	Items	Sources
External	Familiarity with high-tech digital tools (Modern)	As a learner, I am most familiar with …	**[60, 61]**
		…(R1) interactive board Interactive learning tool–new generation	
		…(R2) Internet of Things (cloud-based service tools like Google Drive, Docs and Earth)	
		…(R3) software like IBM SPSS® software	
		…(R4) online course materials*	
		…(R5) e-textbooks*	
		…(R6) smartphones and tablets*	
	Familiarity with classical digital tools (Traditional)	As a learner, I am most familiar with …	
		…(R8) audio and video equipment	
		…(R9) digital projectors	
		…(R10) interactive exercises, games, and presentations	
		…(R11) laptop or computer	
Mediating	Perceived barriers (Barrier)	The digital tools' usage in education is obstructs by…	**[62]**
		…. (CR1) costs of different digital tools	
		…. (CR2) uncertainties related to the different digital tools' quality	
		… (CR3) too much time spend for learning to use its	
		… (CR4) lack of awareness of intellectual property	
		… (CR5) lack of proper digital competence	
	Computer anxiety (Anxiety)	(AT1) Working with digital tools makes me nervous.	**[37]**
		(AT2) Digital tools give me an unpleasant feeling.	**[63]**
		(AT3) Digital tools make me feel uncomfortable.	**[51]**
Internal	Perceived usefulness (Usefulness)	(OR1) Digital tools use can improve my knowledge exchange.	**[27]**
		(OR2) Digital tools use can enhance self-education.	
		(OR3) Digital tools use would allow me to complete homework more quickly.	
		(OR4) Digital tools use can increase my learning performance.	
		(OR5) Digital tools use can increase my learning efficiency.	
	Perceived Ease of Use	(PEU1) I find digital tools to be easy to use from anywhere.	**[1, 64]**
		(PEU2) Using any digital tools is clear and logical.	
		(PEU3) Digital tools provide flexibility in interaction with the user*	
		(PEU4) I could easily acquire useful skills needed to use any digital tools*	
		(PEU5) I find digital tools to be easy to use anytime*	
		(PEU6) I can use any digital tools without problems if I have support*	
	Behavioral Intention to Use	(BU1) Assuming I have permission to use, I will use different digital tools.	**[58]**
		(BU2) I will use different digital tools to search for data, if necessary.	
		(BU3) I intend to use different digital tools, but after I documented.	

* Eliminated during factor analyses.

This structure of the scale was further analyzed and confirmed during validation and cross-validation tests in Stages III and IV. Factor loadings and the internal consistency of each dimension were ensured by the Cronbach's alpha values, which ranged from 0.736 to 0.917. Their values are presented in S5 Appendix.

The factorial structure of seven dimensions explained 71.288% of the total variance. The factor loading of the distinct items oscillated from 0.551 to 0.943, and the explained variances varied from 3.156% to 34.107%. For this part of the study, KMO Measure of Sampling Adequacy and Bartlett's Test of Sphericity (BTS) was found to be 0.875. All the dimensions had robust internal consistency by means of the Cronbach's alpha values around a total scale value of 0.901. Thus, substantial reliability and internal consistency among items in the factorial structure were established.

### Measurement model results

#### Confirmatory factor analysis results

The factor structure resulted in Stage II was validated using confirmatory factor analysis (CFA) for Sample III (N = 262), Sample IV (N = 310), and also for Total Sample (N = 572) composed of both Samples III and IV. Each of these samples was used to test if the theorized structure provided a good-fitting measurement model. The final CFA outputs suggested an adequate measurement model fit for the seven-dimensional 25-item measurement scale for each sample and for the overall sample.

**Fig 1:**
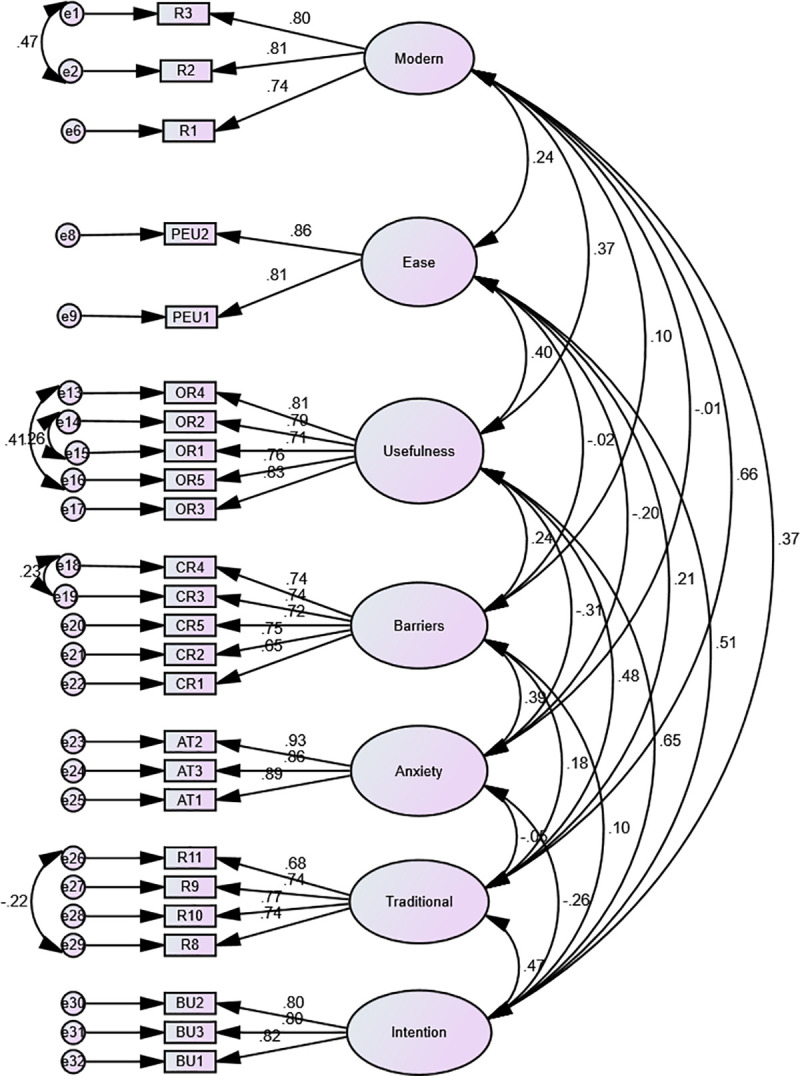
CFA (1) model that reproduces relationships among theoretical constructs. Study case on the overall sample (N = 572).

Confirmatory factor analysis results corresponding for Total Sample are presented in Fig 1. As shown in Fig 1, all factor loadings of the seven dimensions ranged from 0.55 to 0.97, exceeding the required standard of 0.50 [84]. Moreover, Fig 1 shows that the confirmed measurement model for the Total Sample highlights the differences between observed and latent variables.

Five fit indices were used to find measurement model fit, i.e., Chi-square/degree of freedom X^2^/df (*criterion chi-squared /df <3*), Tucker-Lewis index TLI (*criterion > 0*.*90*), comparative fit index CFI (*criterion > 0*.*90*), root mean square error of approximation RMSEA (*criterion < 0*.*08*), and Standardized Root Mean Square Residual SRMR (*criterion < 0*.*08*) [85–87]. More details related to the five fit indices corresponding to each sample are presented in Table 3. It could be observed that all fit indices of the measurement models meet the quality thresholds. So, the confirmatory factor analysis shows that the seven-dimensional 25-item measurement scale presents fit indices that are suitable for the Total Sample as well as for the Sample III and Sample IV [27].

**Table 3:** Summary of CFA models fit indices corresponding to Sample III (N = 262), Sample IV (N = 310), and Total sample (N = 572).

Model	χ2/df	TLI	CFI	RMSEA (90% CI)	SRMR
CFA (Sample III)	1.905	.934	.945	.059	.047
CFA (Sample IV)	2.083	.923	.936	.059	.056
CFA (Total Sample)	2.029	.961	.968	.042	.040

#### Construct validity

S6 Appendix lists the convergent and discriminant validity coefficients corresponding to Sample III, Sample IV and Total Sample. As clearly shown in S6 Appendix, the calculated values of convergent and discriminant validity coefficients exceeding the threshold values [88]. The squared multiple correlations between the research dimensions correspond to the highlighted diagonal elements (in bold). The composite reliability (CR) ranges from 0.779 to 0.941 (Sample III), from 0.821 to 0.912 (Sample IV), and from 0.822 to 0.925 (Total Sample). The average variance explained (AVEs) ranges from 0.552 to 0.843 (Sample III), from 0.500 to 0.777 (Sample IV), and from 0.528 to 0.803 (Total Sample) (S6 Appendix). These outcomes indicate that all three models meet the criteria of convergent validity. As shown in S6 Appendix, the diagonal values range from 0.743 to 0.918 (Sample III), from 0.706 to 0.882 (Sample IV), and from 0.727 to 0.896 (Total Sample), suggesting that all the dimensions of the model have proper discriminant validity.

### Structural model results

**Fig 2:**
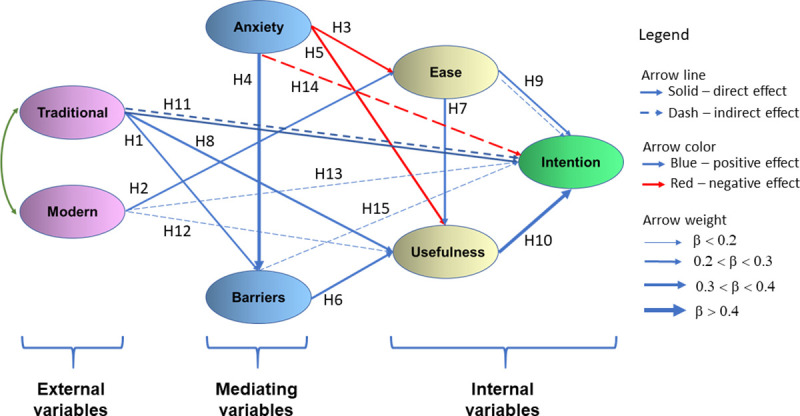
Path model (1) highlighting the significant direct link between factors. 262 undergraduate students with Bachelor's in psychology.

**Fig 3:**
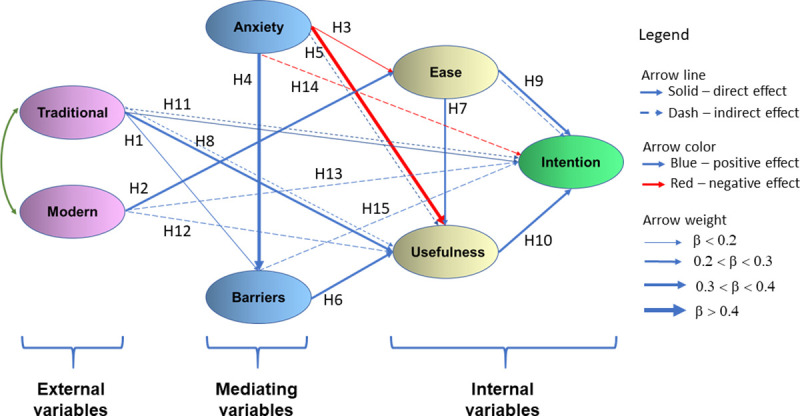
Path model (2) highlighting the significant direct link between factors. 310 master's students in Educational Science and Environmental Science.

Once a suitable fit of the measurement model was demonstrated, the structural invariance tests were completed. In the present study, a structural equation modelling (SEM) technique was performed to estimate the link between each dimension of the BLS scale and student's acceptance of digital learning tools in higher education in a blended learning context. The structure of the path diagram (Figs 2 and 3) indicates the relationships between the dimensions, according to the tested hypotheses. In this way, it is possible to visualize both the direct and indirect effect that one factor has on others. Like the measurement model, the same five fit indices were used to find structural model fit for each data. The results showed that the all fit indexes examined in the case of the hypothesized structural model were fulfilled according to quality index values [89].

For undergraduates enrolled in Psychology study program (N = 262) the structural equation modelling results indicated an acceptable level of the model quality: χ2/Df = 3.157, TLI = 0.948, CFI = 0.978, RMSEA = 0.091, SRMR = 0.048. For graduates (master's students) enrolled in Education Science and Environmental Science study programs (N = 310) structural equation modelling the results indicated a good level of the model quality: χ2/Df = 1.678, TLI = 0.984, CFI = 0.993, RMSEA = 0.047, SRMR = 0.047. As can be observed, the SRMR vary from 0.048 to 0.047, indicating that the structural model satisfactorily demonstrated the model fit [90].

The best path models predicting the intention to use DETs, perceived their usefulness and ease of use among undergraduates and master's students are represented in Figs 2 and 3. Both path models included the moderating effects of perceived barriers and computer anxiety.

The direct effects, indirect effects, total effects and t-value according to each of the three endogenous latent variables, Behavioral intentions to use (Intention), Perceived usefulness (Usefulness) and Perceived easy to use (Ease), and two exogenous variables, Familiarity with high-tech tools (Modern) and Familiarity with traditional tools (Traditional) mediated by Perceived barriers (Barriers), Computer Anxiety (Anxiety), were estimated. The results of conducted path analysis for study case on 262 undergraduates students in Psychology, UB (Path (1) model) and study case on 319 master's students in Educational Science and Environmental Science, UVABc (Path (2) model) are accessible in Table 4. The structure of the path diagram indicates the relationships between the dimensions, according to the tested hypotheses. This allows the user to decide how to select items and checks how responses are correlated (Table 4). Note that each hypothesis is associated with examining the relationship between two model dimensions (Table 4). For example, in the case of hypothesis H1, the relationship between Traditional and Barriers was examined and for hypothesis H2, the relationship between Modern and Ease was examined. Significant total effects have been found across both study cases. Hence, all hypotheses are supported.

**Table 4:** The results of conducted path analysis for study case III on 262 undergraduates students in Psychology, UB (path (1) model) and study case IV on 310 master's students in educational science and environmental science, UVABc (path (2) model).

Hypothesized path	Study case III				Study case IV			
	*Standardized causal effects for the model*			t-value	*Standardized causal effects for the model*			t-value
	Direct effect	Indirect effect	Total effect		Direct effect	Indirect effect	Total effect	
Traditional→Barriers (H1)	0.290		0.290	5.422**	0.167		0.167	3.392*
Modern→Ease (H2)	0.273		0.273	4.812**	0.314		0.314	5.890**
Anxiety→Ease (H3)	-0.293		-0.293	-5.163**	-0.150		-0.150	-2.807*
Anxiety→Barriers (H4)	0.410		0.410	7.670**	0.472		0.472	9.578**
Anxiety→ Usefulness (H5)	-0.337	0.050 (NS)	-0.287	-7.023**	-0.473	0.137*	-0.337	-10.682**
Barriers→Usefulness (H6)	0.346		0.346	7.175**	0.379		0.379	8.515**
Ease→Usefulness (H7)	0.315		0.315	7.060**	0.283		0.283	7.020**
Traditional→Usefulness (H8)	0.372		0.372	8.272**	0.364	0.063*	0.427	8.982**
Ease→ Intention (H9)	0.212	0.180*	0.392	5.583**	0.398	0.103*	0.501	9.331**
**Usefulness→ Intention (H10)**	0.572		**0.572**	12.970**	0.365		**0.365**	7.661**
Traditional→ Intention (H11)	0.236	0.270**	0.506	5.919**	0.175	0.156*	0.331*	3.954**
Modern→ Usefulness (H12)		0.080*	0.080*			0.089*	0.089*	
Modern→ Intention (H13)		0.107*	0.107*			0.157*	0.157*	
Anxiety→ Intention (H14)		-0.227*	-0.227*			-0.182*	-0.182*	
Barriers→ Intention (H15)		0.198*	0.198*			0.138*	0.138*	

NS = Not significant;

*p < .05;

**p < .001

Among the seven dimensions that exhibited significant relationships of Path (1) model, *Perceived usefulness* showed a substantial direct effect on *Behavioral intentions to use* (β = 0.572). *Familiarity with traditional tools* (β = 0.236,) had positive direct effects on *Behavioral intentions to use*, while *Anxiety* had only an indirect negative effect (β = -0.227,) on the same dependent variable. Also, *Familiarity with traditional tools* had strong positive indirect effects (β = 0.270), *Familiarity with modern tools* had weak positive indirect effects (β = 0.107), *Perceived ease of use* (β = 0.189) and *Perceived barriers* (β = 0.198) had positive indirect effects on Behavioral intentions to use.

According to the Path (2) model, the dimension of the *Perceived ease of use* (β = 0.501) had the strongest total effect on *Behavioral intentions to use*, the dimension of *the Perceived usefulness* (β = 0.365) had the second strongest total effect and *Familiarity with traditional tools (Traditional)* (β = -0.331) had the third strongest total direct effect on the main dependent variable, Behavioral Intention to use DETs (Table 4). The results of the Path (2) model are similar to results of the Path (1) model.

As shown in Table 4 and Figs 2 and 3, among external variables the traditional tools showed the strongest total effect (β = 0.506, p < 0.001 for Stage III, and β = 0.331, p < 0.05 for Stage IV), suggesting that modern tools do not have the same effect on behavioral intention to use it. More precisely, the familiarity with traditional tools (*Traditional*), as opposed to the familiarity with high-tech tools (*Modern*), has significantly influenced, both direct and indirect, the intention of adoption of digital tools in a blended learning context, independent by the degree level of students. The familiarity with high-tech tools (*Modern*) has only an indirect influence on the intention to use DETs. Moreover, the results of both path analyses reveal strong negative direct and indirect effects of the *Anxiety* dimension on the *Usefulness* dependent variable.

#### Structural invariance model results

Once an adequate fit of the structural model for all data was found, the invariance procedure was utilized. So, the multigroup analysis of structural invariance was used to test the invariance across degree levels (i.e., Bachelor's versus Master's degree) of university students. The results showed that the all five fit indexes examined in the case of the hypothesized structural invariance model, were fulfilled according to quality index values: χ2/df = 2.418, CFI = 0.986, TLI = 0.967, RMSEA = 0.050, SRMR = 0.048. These outputs confirmed that configural invariance along degree levels was reached [71]. Thus, the pattern of parameters in the hypothesized structural model was identical for the undergraduates and master's students. The structural invariance of the scale was supported because the differences Δχ^2^ (Δdf) between structural weights of Sample III and IV (*Δχ^2^ of 19*.*495 with eleven degrees of freedom*) was not statistically significant at *p* = 0.05 [71].

A Chi-square difference test was run to test the multi-group effects of student degree levels for each hypothesized relationship from Table 1. For example, accordingly with H14 was tested if the negative relationship between computer anxiety the behavioral Intention to use was similar for students enrolled in Bachelor's degree program and for students enrolled in Master's degree programs. No significant differences were found along all tests according to the assumptions mentioned in Table 4, except for the positive relationship between perceived usefulness and behavioral Intention to use DETs. The results of multi-group analyses revealed that students enrolled in Bachelor's degree program have a stronger relationship between Usefulness and Intention than those enrolled in Master’s degree programs.

In the conducted path analysis, all seven factors demonstrated significant total effects on student's acceptance of digital learning tools in higher education in a blended learning context. According to the path analysis results (Table 4), all hypotheses were accepted. Finally, 62% of the variance of the dependent variable, intention to use DETs, was explained by seven-dimensional 25-item survey. Overall, the results of the measurement and structural models indicated a close fit and facilitated to appreciate the causes of the relationship among constructs. Therefore, it can be concluded that the proposed aim of the research has been successfully achieved.

## Discussion

Many researches have indicated that the previous ICT experience of students is an important antecedent of behavioral intention to use it. Consequently, many authors have examined the impact of a different kind of learners’ ICT experience on digital tool adoption. Otherwise, the blended learning is a challenge for both students and higher education teachers. Despite this, there is no brief measurement tool that provides valid and reliable understandings of blended learning adoption by different kind of students in higher education. In this context, in-depth researches are needed to define the relationships between the predictors (i.e., degree of familiarity with traditional and modern technology), the mediators (i.e., perceptions of barriers and the anxiety manifested by users), and the respondent variables (i.e., perceived ease of use, perceived usefulness and behavioral intention to use digital tools), in a mixed learning process, for each degree level (i.e., Bachelor's and Mater's degree levels). From this perspective, it was considered appropriate to investigate the relationships between dependent variables presented in the literature as a measure of the modern technology acceptance and familiarization with traditional and modern digital resources, mediated by psychological factors. By proposing a reliable and valid tool to measure the adoption of digital tools in the blended learning context in higher education, the gaps in existing literature were field.

Briefly, the primary purpose of this study was to develop, validate and test a simple, but reliable and invariant scale useful to evaluate and measure the relationships between familiarity, the perception of barriers and anxiety, the perception of utility, the perception of ease to use, and intention to use DETs. Exploratory Factor Analyses, Confirmatory Factor Analyses and Structural equation modelling were used as the main methods of analysis in this research together with the multi-group analysis of invariance who was performed on the two independent samples (i.e., Bachelor's and Master's degree level).

Fifteen hypotheses were formulated to determine the relationship between various dimensions in the structural model (Table 1). The hypotheses were tested by investigating the path coefficients (Table 4).

The results of convergent and discriminant validity confirmed the construct validity of the measuring instrument (S6 Appendix). Therefore, it can be assumed that the measurement model fits the data properly, so the model can be used to analyze the research hypotheses. The experimental results of this research confirmed all hypotheses suggested for this study.

Thus, the familiarity with traditional tools (audio and video equipment, digital projectors, interactive exercises, games, and presentations or video projectors, games, presentations and interactive face-to-face exercises, computer/laptop) used in face-to-face teaching during Bachelor’s or Master’s degree has a total effect on the intention for use DETs by students. This output does not justify the influence of familiarity with traditional tools on digital tools acceptance; it also explores how this dimension indirectly influences the digital tools adoption in blended learning context. For both degree levels, this study confirms the mediating role of the usefulness between the familiarity with traditional tools and digital tools acceptance. It is interesting to mention that the mediating role of the perceived usefulness concluded in this study supports the view of Rakoczy and collaborators [91]. The utility perception of DETs plays an important positive role for all users, regardless of the degree level they are enrolled in, as opposed to anxiety, which also has a significant but negative direct contribution to the model. Unlike the traditional tools, the modern ones (interactive boards, services based on cloud software or scientific software) have only an indirect, but statistically significant influence on the intention of use, the level of familiarity with modern tools being about 20% lower than in the case of the traditional ones. The familiarity with traditional digital tools leads the student, regardless of the degree level, to adopt DETs resources in blended learning. Familiarity with modern digital resources has only an indirect influence on the intention of use, the perception of ease of use having also a mediating role, as shown by Davis and Wiedenbeck [92]. Through the path analysis, this research validated the mediator role of computer anxiety in digital tools adoption in a blended learning context. These findings are in agreement with the results of Mac Callum and collaborators [93] in case of mobile learning, and by with the results of Cazan and collaborators in case of computer and Internet use in Romanian high-school and university students [63].

In summary, only those modern resources that are perceived as easy to use will be adopted by both undergraduate and master’s students. The utility is a main factor of influence of the intention of use, independent of the university degree level of the learner, a result similar to that published by Abdullah and collaborators [37]. Ease of use influences directly and indirectly the intention of use of all students. The anxiety mediator factor had a significant direct and indirect negative influence on the intention of use. This result is similar to most psychological research that indicates a negative correlation between anxiety and cognitive performance on tests [94, 95]. The anxiety mediator factor had a significant direct negative influence on the perception of utility for all students, regardless of the level of education. However, only in the case of master's students, indirect influence of anxiety on the perception of the utility, through the perception of barriers, could be highlighted. The perception of barriers has significantly influenced the perception of the utility, both for undergraduate and master's students. An essential difference was noted between the master's students and undergraduate students in the relationship between the intention to adopt DETs and the utility. The link between the intention to adopt digital technologies in a mixed learning context and the perception of their usefulness is much stronger for undergraduate students than for those enrolled in the master's program. Specifically, regarding the intention to adopt digital technologies in a mixed learning process, which implies the use of both groups of educational resources, those intended for "face-to-face" learning simultaneously with those of e-learning. Ease of use directly and indirectly influences the intention to use of all students. Users' perceptions of utility and ease to use digital tools have been shown by various research to be critical factors that influence the adoption of digital technologies in education [71]. These conclusions are similar to those obtained in this research. The contribution made by present study consists of the construction of a simply but reliable and valid tool, which can be used to test the level of adoption of technology by undergraduate and master’s students. Small but essential differences between degree levels related to intention to use DETs could be detected in the tests carried out. It was found that the perception of DETs utility influences more undergraduate students than master’s students in terms of the intention to adopt digital resources. Possible explanations comprise the exclusion of other important variables in the model, which are more sensitive to students enrolled at a higher level of education, such as previous experience of using digital resources in the context of blended learning. Another explanation can be the difference in attitude between the two types of students: the students of the bachelor's degree want to learn many things from different domains; the master students are focused on specific competencies.

## Implications for researchers and educators

According to the results of this research, several recommendations can be proposed for researchers and educators to increase the intention to use DETs by students in higher education:

- identifying solutions to increase the degree of familiarity with modern tools;
- identifying solutions to reduce digital anxiety;
- enhancing access of students to DETs;
- providing clear and logical instructions for DETs users in order to quickly learn the steps required for using its;
- identifying solutions to reduce the cost for DETs;
- quality assurance of DETs who are recommended to be used;
- reducing the time required to be invested for DETs use;
- promoting the need to recognize intellectual property rights in a digital environment;
- promoting the need to acquire digital skills for all DETs users.

The results of this study showed a more significant contribution of Tradition than Modern digital tools to the hypothetical research model. It is a natural thing, which confirms the rigor of the investigation, but in the context of promoting mixed learning, familiarity with each category of resources plays an important role. The predominant use of traditional resources increases the barriers perceptions that induces a state of anxiety, which is not evident in the case of the preferential use of modern resources.

The intention to use mixed resources is influenced by the following factors, ordered according to the intensity of the contribution to the Intention to use DETs:

- For undergraduate students: Utility > Traditional > > Ease of Use;
- For master’s students: Ease of use > Utility > Traditional resources.

A significant negative contribution of the Anxiety factor was observed on all internal variables: the intention of use DETs for all students, perceived utility, and ease of use, but the most substantial relationship is with Utility factor. These results can be explained if the anxiety is appreciated as a personality trait [96] that generally influences human behavior. Usually, the level of a bachelor's degree is seen as more important than the master's degree. As a result, students from undergraduate studies develop higher anxiety about the educational resources because the stake for the successful completion of a bachelor's degree program is higher. On the other hand, the learning style in master's programs is more applicable to collaborative topics of greater interest, motivation is higher, the topics addressed are learned with pleasure. One possible conclusion is that the adoption of digital learning tools is influenced by personality factors that develop differently in particular learning contexts.

The results obtained from the application of this new instrument, developed, validated and tested in this study, contribute to highlighting the student behavioral differences, from the perspective of their intention to use DETs in a mixed learning context.

## Limitations and future directions

The research has some limitations. The small sample size for Confirmatory Factor Analyses and the use of only one sample for external validation are the main limitations. The small sample size was inevitable because of the necessity to use students from the same categories [97]. But future studies will take account of these aspects and the external validation will be extended. Despite the potential high correlation between particularities of each country and adoption of innovative educational resources is planned to compare the results of this extended TAM model from one country inside the European Union (Romania) and another one outside UE (South Africa). These future studies will contribute to understanding possible challenges and barriers thus making the internationalization of the study.

## Conclusions

There is large research about digital technologies' adoption in the educational field. The diversity of outcomes obtained by previous studies conducted on different target audiences in traditional educational background support the usefulness of the research in other educational contexts than the classical ones. The research data about learners' preferences for specific digital technologies is difficult to ascertain and more difficult to build a universal research instrument applicable to test acceptance of digital resources for blended learning instruction. The adoption of digital technology is influenced by many external variables among which user's familiarity is not a well-explored one. Moreover, no studies have been conducted on the correlation between *familiarity with digital tools* and *acceptance to use them*, mediated by computer anxiety. In many countries, the progress of technology to enhance teaching, learning, and collaboration has become gradually important over the last decade. Thus, a better understanding of learners' acceptance and usage behavior of different kind of digital tools in universities is fundamental in increasing future considerations and utilization of new educational technologies, particularly in a blended learning context. The novelty of the study is the development of an extended Technology Acceptance Model that takes into account the influence of two related predictors—familiarity with traditional and modern tools on the intention to use them, in a blended learning context. The influence of familiarity with traditional instruments is significant on the intention to use as opposed to familiarity with high-tech instruments that have a moderate indirect effect but statistically significant.

Results of this research contribute to current literature on the adoption of digital tools in a blended learning context. This study produces new information that differentiates the BLS scale from other instruments used for technology adoption exploration. Study outcomes indicated that the research tool can be used for exploring the key factors influencing blended learning adoption in higher education. Exploratory factor analysis and Confirmatory factor analysis determines whether the hypothesized structure be responsible for a good fit to the data and confirms the survey construct validity. To better appreciate the causes of association among constructs, also a path analysis was performed. The CFA confirms the seven dimensions structure of BLS with three endogenous latent variables (Behavioral intentions to use, Perceived usefulness and Perceived easy to use), two exogenous variables (Familiarity with high-tech tools and Familiarity with traditional tools), and two mediator variables (Perceived barriers, Computer Anxiety). Only one dimension (Computer Anxiety) have negative antecedents.

The overall results indicate that the BLS has appropriate validity and reliability. The BLS scale is also invariant across education level groups. Results on multi-group path analysis have demonstrated a stronger relationship between perception of DETs utility and intention to use them for students enrolled in undergraduate program other than those enrolled in master program. Findings of the current research recommend the necessity to be made available to students educational software, interactive board and scientific software for a longer period of time, to increase the degree of familiarity with their use and implicitly will increase their interest in using its. Having good psychometric properties, the BLS can be used in the next studies for the improvement of teaching-learning strategies in a blended learning context.

## Supporting information

S1 Appendix Studies used to develop the initial pool of items. (DOCX)

S2 Appendix Synthesis of the procedures used to develop the instrument. (DOCX)

S3 Appendix Questionnaire used in this study. (PDF)

S4 Appendix Data set. (XLSX)

S5 Appendix The results of the analysis of the questionnaire regarding mean, standard deviation, skewness, kurtosis, corrected item-total correlation for each item and Cronbach's alfa coefficient of the scores of each subscale from Stage I (n = 250). (DOCX)

S6 Appendix Convergent and discriminant validity coefficients corresponding to Sample III, N = 262 (a), Sample IV, N = 310 (b) and total sample, N = 572 (c).
(DOCX)
