# Supplementary material for: Digital technology adoption scale in the blended learning context in higher education: Development, validation and testing of a specific tool
Source: PLoS One. 2020 Jul 10;15(7):e0235957. doi: 10.1371/journal.pone.0235957 (PMC7351189; doi:10.1371/journal.pone.0235957)
Supplement: S1 Appendix — (DOCX) [file pone.0235957.s001.docx]

**S1 Appendix.** **Studies used to develop the initial pool of items.**

| **DETs** | **Context**  **/Country** | **Research model** | **External variables** | **Mediating and response variables** | **Target group size** | **References** |
| --- | --- | --- | --- | --- | --- | --- |
| e-learning;  e-portfolios | Firstly, the study reviewed the existing literature in e-learning adoption by users and examined the relationship between variables. Secondly, the study was applied for testing e-portfolios adoption in a large university from UK. | General Extended Technology Acceptance Model for E-Learning (GETAMEL) | Self-Efficacy, Subjective Norm, Enjoyment, Computer Anxiety and Experience | Perceived Ease of Use, Perceived Usefulness and  Behavioural Intention to use | undergraduate students from a large UK university (n=242) | [1, 2] |
| mobile learning | Research on effects of quality features on mobile learning acceptance in Jordan | Extending technology  acceptance model (TAM) and the updated DeLone and McLean information  system success mode | Learning content quality  Content design quality  Interactivity  Functionality  User-interface design  Accessibility  Availability  Personalization  Responsiveness | Perceived Ease of Use, Perceived Usefulness and  Behavioural Intention to use | students from five universities (n=400) | [3] |
| e-learning | Research regarding causes of e-learning success in Portugal | DeLone & McLean IS success model and Individualism/Collectivism model | User Satisfaction (US);  Effective use | Individual student’  perception of the organizational impacts/ Individualism/Collectivism | students from 11 universities (n=397) | [4] |
| online  learning technologies | Research about using online learning technologies in vocational education context in Canada | TAM model | Attitude towards use | Perceived Ease of Use, Perceived Usefulness and  Behavioural Intention to use | 213 students from pre-university science College | [5] |
| television and online video platform | Research about the factors who influence audiences for online video, and some television websites in USA | Theory of planned behaviour (TPB) and the technology acceptance model (TAM) | Perceived  characteristics of  online video platforms and Consumer characteristics | Intention to use  online video platforms;  Intention to use  television | 1500 adults who use the Internet | [6] |
| mobile learning | Research about implementing of m-learning in developing countries in Iran | Technology Acceptance Model (TAM) and Unified Theory of  Acceptance and Use Technology (UTAUT) | Pedagogical factors (learning content quality, interactivity); Technological factors (facilitating conditions, user interface and mobile device limitations); Social factors (government support, social influences); Individual factors (Personal Innovativeness, Self-efficacy, Trust) | Perceived Ease of Use, Perceived Usefulness and  Behavioural Intention to use m-learning | undergraduate, graduate and postgraduate  students (n=257) | [7] |
| YouTube | Research about measuring of behavioural  intentions to use YouTube as a Learning Resource in India | Technology Acceptance Model (TAM) | Not applicable | Perceived Usefulness, Perceived Ease of Use, User Attitude, and Behavioural Intention to use | students, research scholars or faculty members (n=140) | [8] |
| laptop | Research on Higher education laptop initiatives/USA | Technology Acceptance Model (TAM) | Not applicable | Acceptance of laptop,  Perceived requirements, Perceived usefulness, Perceived ease of use, and Perceived change | undergraduate and graduate students enrolled in a mid-sized four-year university (n=272) | [9] |
| Facebook | To study in which conditions profiles of motivation can be identified in Romania | Latent profile analysis  (LPA) and mclust package version 5.3 in R | Not applicable | Maintaining social relations (MSR), extending  social relations (ESR), and information &  collaboration (IC) | one sample (n=557) was used to identify Romanian university students’ motivational profiles and other sample (n=550) was used to replicate the motivational configurations and cross-validate results found in first study | [10] |
| e-portofolio | Research on resources for staff to enhance their teaching effectiveness  in higher education in Hong Kong and Taiwan | Technology Acceptance Model (TAM) | Concern about time, Concern about Technology and Support Efficacy in using DTP by Self-Exploration,  Efficacy in using DTP with Professional Guidance | Personal Benefits,  Social Benefits,  Ease of use,  Intention to use | teaching  staff from two tertiary institutions (n=132) | [11] |
| online social  networking | popularity of social technologies/Malaysia and Australia | Focus group discussions; All interview transcripts were printed, read multiple times, and notes were recorded in the margins to identify potential themes | - | - | students from Malaysian  and Australian universities (n=414) | [12] |
| Web 2.0 applications | Research on how learners utilize these applications for learning in USA | Unified Theory  of Acceptance and Use of Technology (UTAUT) | - | performance  expectancy (PE) attitude (ATUT), and anxiety (ANX) | students from a public university (n=432) | [13] |
| Interactive whiteboard | Study the effect of IWB-assisted courses on learners in Turkey | Constructivism Theory and the  Technology Acceptance Model | Not applicable | Perceived Learning Contribution and Motivation, Perceived Efficiency, and Perceived Negative Effects | middle school students from Istanbul, Turkey (N = 263) | [14] |
| e-book | Korea | TAM | Compatibility (COM)  Relative advantage (RA)  Self-efficacy (SE)  Subjective norms (SN) | Perceived ease of use, Perceived usefulness,  Satisfaction with e-book,  Intention to continue using | College students  n = 1030 | [15] |
| Web 2.0 applications  (blog, wiki, social networking tool, online video sharing tool, online game, and immersive virtual environment) | USA | Unified Theory  of Acceptance and Use of Technology (UTAUT) | Performance expectancy (PE), effort expectancy (EE), social influence  (SI), and facilitating conditions (FC) | Performance expectancy (PE), effort expectancy (EE), social influence  (SI), facilitating conditions (FC), attitude toward using technology (ATUT), self-efficacy (SE), anxiety (ANX),  users' behavioral intentions (BI) and actual user behaviors (UB) | college students from one public  university in the United State (n=432) | [13] |
| mobile learning | USA | TAM | User interface  Personal innovativeness | Perceived usefulness Perceived ease of  use  Intention to use Satisfaction in learning | 350 students who enrolled in major  courses of W Cyber University | [16] |
| ICT tools | Singapore | TAM | - | perceived  ease of use, perceived usefulness, attitude toward use, and intention to use | 737 first-year polytechnic students | [17] |
| Internet | Taiwan | Social cognitive theory | Self-efficacy beliefs specifically  formed within the academic domain and internet anxiety |  | 12-grade  Students from industrial vocational high  school in Taipei, Taiwan (n=212) | [18] |
| online learning community | Taiwan | TAM | Online Course  Design  User Interface  Design  Previous Online  Learning  Experience | Perceived  ease of use, Perceived usefulness, Perceived  Interaction,  Intention to Use an Online  Learning  Community | 436 Taiwanese senior high school students | [19] |
| e-learning | Iran | TAM and D&M | system, service, information quality,  educational quality | Satisfaction,  Intention to use, Perceived usefulness and Perceived ease  of use | 390 students from 4 university | [20] |
| online learning environments | Turkey | Technology Continuance  Theory, Information Systems Success Model, Cognitive Model and Information Systems Expectation  Confirmation Model |  |  | 467 public university students | [21] |
| Facebook | Serbia |  | Frequency of Facebook wall use. |  | University of Belgrade e  Faculty of Transport and Traffic Engineering | [22] |
| digital teaching portfolio | Hong Kong and Taiwan | Correlation analysis,  independent sample t-test and one-way ANOVA | Concern about Time, Concern about Technology and Support, and Computer Efficacy |  | 132 teaching  staff from two tertiary institutions from Hong Kong and Taiwan | [11] |
| interactive whiteboard | Turkey | Unified Theory of Acceptance and Use of Technology UTAUT theory | Performance expectancy, effort expectancy, social influence and facilitating conditions; gender, age, experience and voluntariness are the moderators |  | Teachers (n=158) | [23] |
| ICT tools | Malaysia | Technology  Acceptance Model with Willing’s learning styles  construct | learning styles on this usage | extent of use, perceived  usefulness and perceived ease of use | 328 questionnaires address to  undergraduates who were taking an English for Academic Purposes (EAP) course | [24] |
| digital technologies in a blended learning context | Uganda | Technology Acceptance Model (TAM) | Students capacity to use digital technologies, Students’ Access to digital technologies,  Students’ Awareness of Use of digital technologies, Lecturer characteristics | Perceived Ease of Use, Perceived Usefulness,  Attitude toward Usage, Intention to Use | 341 students doing their undergraduate  programs | [25] |
| Web 2.0 | USA | Structural Model | attitudes toward learner  self-direction, instructional technology, and innovation; external facilitators and constraints; Web 2.0  knowledge and interest; | intended and actual use of Web 2.0 | online instructors at the selected institutions (n=285) | [26] |
| MOOCs | China | technology  acceptance model (TAM) and task technology fit (TTF) model | TTF model, features of MOOCs, and social motivations | perceived usefulness  perceived ease of use | 252 valid surveys were returned. And all the respondents were Chinese from tier cities in China | [27] |
| multimedia content | USA | integrated model, which extends the technology acceptance  model TAM with task technology fit TTF theories | Technology characteristics, Task technology fit and Prior Experiences;  a single-item measure for individual  characteristics (prior experience) was used |  | Students from three  south-eastern universities, USA (n= 120) | [28] |
| e-books | Korea | Hybrid model integrating the Theory of Reasoned Action TRA, the Theory of Planned Behavior TPB, and the Diffusion of Innovation Theory (DIT) into the TAM | compatibility, relative advantage of e-book compared to paper books, self-efficacy, and subjective norms |  | Students from two universities in Korea (n = 1030) | [15] |
| e-learning | Lebanon | extended Technology Acceptance Model (TAM) | subjective norms (SN) and Quality of Work Life (QWL) | perceived usefulness  (PU), perceived ease of use (PEOU) behavioural intention (BI)  Actual usage | two universities  located in Beirut (n=596) | [29] |
| e-books | South  Korea | Extended technology acceptance model | perceived playfulness  and risk |  | Students from private education agency  (n=153) | [30] |
| course videos; course documents; exercise | Research about the role of technologies in the academic achievement/Turkey | TAM model | anxiety, facilitative conditions, self-efficacy, attitude, satisfaction and subjective norms | Perceived Ease of Use (PEOU), Perceived Usefulness (PU),  and  Perceived behavior control (PBC)  Continued intention and Achievements | Students from Vocational Higher School (n=500) | [31] |

**References**

1. Abdullah F, Ward R. Developing a General Extended Technology Acceptance Model for E-Learning (GETAMEL) by analysing commonly used external factors. Computers in Human Behavior. 2016;56:238-56. doi: <http://dx.doi.org/10.1016/j.chb.2015.11.036>.

2. Abdullah F, Ward R, Ahmed E. Investigating the influence of the most commonly used external variables of TAM on students’ Perceived Ease of Use (PEOU) and Perceived Usefulness (PU) of e-portfolios. Computers in Human Behavior. 2016;63:75-90. doi: <https://doi.org/10.1016/j.chb.2016.05.014>.

3. Almaiah MA, Jalil MA, Man M. Extending the TAM to examine the effects of quality features on mobile learning acceptance. Journal of Computers in Education. 2016;3(4):453-85. doi: 10.1007/s40692-016-0074-1.

4. Aparicio M, Bacao F, Oliveira T. Cultural impacts on e-learning systems' success. The Internet and Higher Education. 2016;31:58-70. doi: <https://doi.org/10.1016/j.iheduc.2016.06.003>.

5. Bazelais P, Doleck T, Lemay DJ. Investigating the predictive power of TAM: A case study of CEGEP students’ intentions to use online learning technologies. Education and Information Technologies. 2018;23(1):93-111. doi: <https://doi.org/10.1007/s10639-017-9587-0>.

6. Cha J. Predictors of television and online video platform use: A coexistence model of old and new video platforms. Telematics and Informatics. 2013;30(4):296-310. doi: <https://doi.org/10.1016/j.tele.2013.01.001>.

7. Chavoshi A, Hamidi H. Social, individual, technological and pedagogical factors influencing mobile learning acceptance in higher education: A case from Iran. Telematics and Informatics. 2018. doi: <https://doi.org/10.1016/j.tele.2018.09.007>.

8. Chintalapati N, Daruri VSK. Examining the use of YouTube as a Learning Resource in higher education: Scale development and validation of TAM model. Telematics and Informatics. 2017;34(6):853-60. doi: <http://dx.doi.org/10.1016/j.tele.2016.08.008>.

9. Elwood S, Changchit C, Cutshall R. Investigating students' perceptions on laptop initiative in higher education. Campus - Wide Information Systems. 2006;23(5):336-49. doi: doi:<http://dx.doi.org.am.e-nformation.ro/10.1108/10650740610714099>

10. Cristescu I, Balog A. Exploring Motives for Using Facebook: A Latent Profile Analysis Studies in Informatics and Control. 2018;27(3):331-8.

11. Fong RW-t, Lee JC-k, Chang C-y, Zhang Z, Ngai AC-y, Lim CP. Digital teaching portfolio in higher education: Examining colleagues' perceptions to inform implementation strategies. The Internet and Higher Education. 2014;20:60-8. doi: <https://doi.org/10.1016/j.iheduc.2013.06.003>.

12. Hamid S, Waycott J, Kurnia S, Chang S. Understanding students' perceptions of the benefits of online social networking use for teaching and learning. The Internet and Higher Education. 2015;26:1-9. doi: <https://doi.org/10.1016/j.iheduc.2015.02.004>.

13. Huang W-HD, Hood DW, Yoo SJ. Gender divide and acceptance of collaborative Web 2.0 applications for learning in higher education. The Internet and Higher Education. 2013;16:57-65. doi: <https://doi.org/10.1016/j.iheduc.2012.02.001>.

14. Türel YK. An interactive whiteboard student survey: Development, validity and reliability. Computers & Education. 2011;57(4):2441-50. doi: <https://doi.org/10.1016/j.compedu.2011.07.005>.

15. Jin C-H. Adoption of e-book among college students: The perspective of an integrated TAM. Computers in Human Behavior. 2014;41:471-7. doi: <http://dx.doi.org/10.1016/j.chb.2014.09.056>.

16. Joo YJ, Lee HW, Ham Y. Integrating user interface and personal innovativeness into the TAM for mobile learning in Cyber University. Journal of Computing in Higher Education. 2014;26(2):143-58. doi: 10.1007/s12528-014-9081-2.

17. Kwok D, Yang S. Evaluating the intention to use ICT collaborative tools in a social constructivist environment. International Journal of Educational Technology in Higher Education. 2017;14(1):32. doi: 10.1186/s41239-017-0070-1.

18. Chen L-Y, Hsiao B, Chern C-C, Chen H-G. Affective mechanisms linking Internet use to learning performance in high school students: A moderated mediation study. Computers in Human Behavior. 2014;35:431-43. doi: <https://doi.org/10.1016/j.chb.2014.03.025>.

19. Liu IF, Chen MC, Sun YS, Wible D, Kuo C-H. Extending the TAM model to explore the factors that affect Intention to Use an Online Learning Community. Computers & Education. 2010;54(2):600-10. doi: <https://doi.org/10.1016/j.compedu.2009.09.009>.

20. Mohammadi H. Investigating users’ perspectives on e-learning: An integration of TAM and IS success model. Computers in Human Behavior. 2015;45:359-74. doi: <http://dx.doi.org/10.1016/j.chb.2014.07.044>.

21. Dağhan G, Akkoyunlu B. Modeling the continuance usage intention of online learning environments. Computers in Human Behavior. 2016;60:198-211. doi: <https://doi.org/10.1016/j.chb.2016.02.066>.

22. Čičević S, Samčović A, Nešić M. Exploring college students' generational differences in Facebook usage. Computers in Human Behavior. 2016;56:83-92. doi: <https://doi.org/10.1016/j.chb.2015.11.034>.

23. Tosuntaş ŞB, Karadağ E, Orhan S. The factors affecting acceptance and use of interactive whiteboard within the scope of FATIH project: A structural equation model based on the Unified Theory of acceptance and use of technology. Computers & Education. 2015;81:169-78. doi: <https://doi.org/10.1016/j.compedu.2014.10.009>.

24. Thang SM, Nambiar RMK, Wong FF, Mohd Jaafar N, Amir Z. A Clamour for More Technology in Universities: What Does an Investigation into the ICT Use and Learning Styles of Malaysian ‘Digital Natives’ Tell Us? The Asia-Pacific Education Researcher. 2015;24(2):353-61. doi: 10.1007/s40299-014-0185-2.

25. Tulinayo P, Ssentume P, Najjuma R. Digital technologies in resource constrained higher institutions of learning: a study on students’ acceptance and usability. International Journal of Educational Technology in Higher Education. 2018;15. doi: 10.1186/s41239-018-0117-y.

26. Ulrich J, Karvonen M. Faculty instructional attitudes, interest, and intention: Predictors of Web 2.0 use in online courses. The Internet and Higher Education. 2011;14(4):207-16. doi: <https://doi.org/10.1016/j.iheduc.2011.07.001>.

27. Wu B, Chen X. Continuance intention to use MOOCs: Integrating the technology acceptance model (TAM) and task technology fit (TTF) model. Computers in Human Behavior. 2017;67:221-32. doi: <http://dx.doi.org/10.1016/j.chb.2016.10.028>.

28. Park C, Kim D-g, Cho S, Han H-J. Adoption of multimedia technology for learning and gender difference. Computers in Human Behavior. 2019;92:288-96. doi: <https://doi.org/10.1016/j.chb.2018.11.029>.

29. Tarhini A, Hone K, Liu X. The effects of individual differences on e-learning users’ behaviour in developing countries: A structural equation model. Computers in Human Behavior. 2014;41:153-63. doi: <https://doi.org/10.1016/j.chb.2014.09.020>.

30. Park B, Chang H, Park SY. Adoption of digital devices for children education: Korean case. Telematics and Informatics. 2018. doi: <https://doi.org/10.1016/j.tele.2018.11.002>.

31. Cakır R, Solak E. Attitude of Turkish EFL Learners towards e-Learning through Tam Model. Procedia - Social and Behavioral Sciences. 2015;176:596-601. doi: <https://doi.org/10.1016/j.sbspro.2015.01.515>.
