# Supplementary material for: Digital technology adoption scale in the blended learning context in higher education: Development, validation and testing of a specific tool
Source: PLoS One. 2020 Jul 10;15(7):e0235957. doi: 10.1371/journal.pone.0235957 (PMC7351189; doi:10.1371/journal.pone.0235957)
Supplement: S2 Appendix — (DOCX) [file pone.0235957.s002.docx]

**S2 Appendix.** **Synthesis of the procedures used to develop the instrument.**

| **Period of sampling** | **Stage** | **Objectives** | **Assessments** | **Methods** | **Sample** | **Results** |
| --- | --- | --- | --- | --- | --- | --- |
| January-June 2017 | Stage I | Development of the Initial BLS scale model | Item development | Qualitative method – systematic review of literature | - | Preliminary model (*121 items*) |
|  |  |  | Content analysis of the initial pool of items contents | Correction based on experts’ opinions | - | Corrected preliminary model (*36 items*) |
|  |  |  | Item analyses *(36 items)* | Descriptive statistics; item-total correlation (ITC), internal consistency (IC) and experts’ opinions | 250 undergraduate students engineering studies, University of Bacau | Initial model (*32 items*) |
|  | Stage II | Identification of the dimensions of the Initial BLS scale model | Dimensionality | Exploratory factor analysis (EFA) | 206 undergraduate students from Education Science studies, University of Bucharest | EFA model (*7 factors with 25 items*) |
| January-June 2018 | Stage III | The goodness-of-fit of data from the CFA (1) model | Evaluation of the latent structure (*7 factors with 25 items*) | Confirmatory factor analysis (CFA (1)) | 262 undergraduate students from Psychology studies, University of Bucharest | CFA (1) indicators which best explains the model (*7 factors with 25 items*) |
|  |  |  | Construct validity | Convergent and divergent validity |  | Convergent validity coefficient (CR) of each construct of CFA (1) model is greater than 0.70 and Average variance extracted (AVE) is greater than 0.50 [36, 54]; AVE greater than Maximum Shared Variance (MSV) [36] |
|  |  |  | Measurement invariance | Configural, metric and scalar invariance |  |  |
|  | Stage IV | Cross-validation of the BLS scale | Evaluation of the latent structure - EFA model (*7 factors with 25 items*) | Confirmatory factor analysis (CFA) | 310 graduate students from Education Science studies and Environmental Science studies, University of Bacau | CFA (2) indicators which best explains the model (*7 factors with 25 items*) |
|  |  |  | Construct validity | Convergent validity |  | CR > 0.70 and AVE > 0.50 |
|  |  |  | Construct validity | Discriminant validity |  | AVE > MSV |
|  | Stage V | Explore the relation between dimensions | Testing the structural invariance of model and multi-group  analysis | Path analysis with Structural Equation Modelling (SEM) and multi-group  analysis of invariance | Total Sample corresponding to Stage III and Stage IV | SEM (1) model reproduced relationships among theoretical constructs  (*7 factors with 25 items*) |
