# Supplementary material for: Digital technology adoption scale in the blended learning context in higher education: Development, validation and testing of a specific tool
Source: PLoS One. 2020 Jul 10;15(7):e0235957. doi: 10.1371/journal.pone.0235957 (PMC7351189; doi:10.1371/journal.pone.0235957)
Supplement: S3 Appendix — (PDF) [file pone.0235957.s003.pdf]

S3 Appendix. The questionnaire

Information sheet

Organization:

The research is designed by academics from the Faculty of Psychology and Education Sciences, University of Bucharest. Before deciding whether to participate, it is important for you to understand why we want to conduct this research and what it is supposed to do, and for this reason we invite you to read the following information carefully.

The purpose of this study is to investigate the predictors of the digital educational resources' acceptance by students / graduates of higher education to improve blended learning practices. Also, the results of this study will be used to enhance individual and institutional performance.

Who is invited to participate?

We invite undergraduate and graduate students who have experienced blended learning to participate in our research on a voluntary basis. It is entirely your decision if you want to participate or not, although we would highly appreciate your contribution. If you agree now, but you change your mind later, you can withdraw from the study. This will not affect you in any way.

What will we have to do?

Once we receive your agreement to participate in the study, please sign the informed consent, and then fill in the 2-page questionnaire in which you answer firstly to some generally questions about your profile.

We estimate that the average answer time for all questions will be 5 minutes.

Ethics

This study is in line with the provisions of the Helsinki Declaration on human subjects participating in research. The use of this tool can not be done without the consent of the authors of the questionnaire. The authors of the research are responsible for clarifying any problem by providing all the necessary information.

Confidentiality

After collecting the data, these will be used exclusively for research purposes. All completed questionnaires will be encoded, which will ensure anonymity. The results will only be presented as group statistics and will be available to specialists. They will be used in scientific papers, conferences, studies in research and development projects.

Understanding

I confirm that I have read and understood the information sheet (*check the respective situation*)

Yes ☐ No ☐

I confirm participation in the study as a volunteer (*check the respective situation*)

Yes ☐ No ☐

**Date of completion (*fill in*):**<br>………………	**University where you are studying / completing (*fill in*):**<br>………………………………………..
**1a. Study program (*fill in*):**<br>………………….	**1b. Level of current education (*check*):**<br>Bachelor's ☐ Master ☐
**3. Gender (*check*):**<br>Feminine ☐ Masculine ☐	**4. Age (*fill in*):**<br>………….

**Scale**	**Completely Disagree**	**Disagree**	**No opinion**	**Agree**	**Completely Agree**
As a learner, I am most familiar with …:					
…(R1) interactive board	1	2	3	4	5
…(R2) Internet of Things (cloud-based service tools like Google Drive, Docs and Earth)	1	2	3	4	5
…(R3) software like IBM SPSS® software	1	2	3	4	5
…(R4) online course materials	1	2	3	4	5
…(R5) e-textbooks	1	2	3	4	5
…(R6) smartphones and tablets	1	2	3	4	5
…(R8) audio and video equipment	1	2	3	4	5
…(R9) digital projectors	1	2	3	4	5
…(R10) interactive exercises, games, and presentations	1	2	3	4	5
…(R11) laptop or computer	1	2	3	4	5
The digital tools' usage in education is obstructs by…					
…. (CR1) costs of different digital tools	1	2	3	4	5

…. (CR2) uncertainties related to the different digital tools' quality	1	2	3	4	5
… (CR3) too much time spend for learning to use its	1	2	3	4	5
… (CR4) lack of awareness of intellectual property	1	2	3	4	5
… (CR5) lack of proper digital competence	1	2	3	4	5
Feelings regarding digital tools usage					
(AT1) Working with digital tools makes me nervous.	1	2	3	4	5
(AT2) Digital tools give me an unpleasant feeling.	1	2	3	4	5
(AT3) Digital tools make me feel uncomfortable.	1	2	3	4	5
(AT4) I feel relaxed when I use digital resources	1	2	3	4	5
Perceived ease of use of digital tools					
(PEU1) I find digital tools to be easy to use from anywhere.	1	2	3	4	5
(PEU2) Using any digital tools is clear and logical.	1	2	3	4	5
(PEU3) Digital tools provide flexibility in interaction with the user.	1	2	3	4	5
(PEU4) I could easily acquire useful skills needed to use any digital tools.	1	2	3	4	5
(PEU5) I find digital tools to be easy to use anytime.	1	2	3	4	5
(PEU6) I can use any digital tools without problems if I have support.	1	2	3	4	5
(PEU7) I am sure I can use any digital educational resource without technical guidance.	1	2	3	4	5
(PEU8) I need user instructions for any digital resource.	1	2	3	4	5
(PEU9) I need help from friends to use any digital resources.	1	2	3	4	5
Perceived usefulness of digital tools usage					
(OR1) Digital tools use can improve my knowledge exchange.	1	2	3	4	5
(OR2) Digital tools use can enhance self-education.	1	2	3	4	5
(OR3) Digital tools use would allow me to complete homework more quickly.	1	2	3	4	5
(OR4) Digital tools use can increase my learning performance.	1	2	3	4	5
(OR5) Digital tools use can increase my learning efficiency.	1	2	3	4	5
Intention to use digital tools					
(BU1) Assuming I have permission to use, I will use different digital tools.	1	2	3	4	5
(BU2) I will use different digital tools to search for data, if necessary.	1	2	3	4	5
(BU3) I intend to use different digital tools, but after I documented.	1	2	3	4	5

Thank you for your contribution!
