# Supplementary material for: Digital technology adoption scale in the blended learning context in higher education: Development, validation and testing of a specific tool
Source: PLoS One. 2020 Jul 10;15(7):e0235957. doi: 10.1371/journal.pone.0235957 (PMC7351189; doi:10.1371/journal.pone.0235957)
Supplement: S5 Appendix — (DOCX) [file pone.0235957.s005.docx]

**S5 Appendix.** **The results of the analysis of the questionnaire regarding mean, standard deviation, skewness, kurtosis, corrected item-total correlation for each item and Cronbach's alfa coefficient of the scores of each subscale from Stage I (n = 250).**

| **Factor** | **Item** | Mean | Std. Deviation | Skewness | Kurtosis | Corrected Item-Total Correlation | Cronbach's alfa coefficient |
| --- | --- | --- | --- | --- | --- | --- | --- |
| **Familiarity with high-tech digital tools (Modern)** | As a learner, I am most familiar with … | | | | | | 0.848 |
|  | (R1) interactive board | 2.86 | 1.346 | 0.131 | -1.093 | 0.591 |  |
|  | (R2) Internet of Things (cloud-based service tools like Google Drive, Docs and Earth) | 3.25 | 1.117 | -0.154 | -0.501 | 0.783 |  |
|  | (R3) software like IBM SPSS® software | 3.12 | 1.208 | -0.087 | -0.777 | 0.731 |  |
|  | (R4) online course materials | 3.8 | 1.005 | -0.46 | -0.452 | 0.504 |  |
|  | (R5) e-textbooks | 3.31 | 1.122 | -0.303 | -0.389 | 0.687 |  |
|  | (R6) smartphones and tablets | 3.24 | 1.411 | -0.286 | -1.179 | 0.501 |  |
| **Familiarity with classical digital tools (Traditional)** | As a learner, I am most familiar with … | | | | | | 0.784 |
|  | (R8) audio and video equipment | 3.47 | 1.057 | -0.193 | -0.599 | 0.533 |  |
|  | (R9) digital projectors | 3.86 | 1.018 | -0.544 | -0.429 | 0.553 |  |
|  | (R10) interactive exercises, games and presentations | 3.66 | 1.076 | -0.323 | -0.705 | 0.697 |  |
|  | (R11) laptop or computer | 3.52 | 1.061 | -0.348 | -0.337 | 0.582 |  |
| **Perceived barriers (Barrier)** | The digital tools’ usage in education is obstructs by… | | | | | | 0.865 |
|  | (CR1) costs of different digital tools | 3.04 | 1.084 | 0.027 | -0.317 | 0.632 |  |
|  | (CR2) uncertainties related to the different digital tools’ quality | 3.21 | 1.139 | -0.022 | -0.652 | 0.680 |  |
|  | (CR3) too much time spend for learning to use its | 3.09 | 1.043 | -0.164 | -0.199 | 0.730 |  |
|  | (CR4) lack of awareness of intellectual property | 3.1 | 1.035 | -0.18 | -0.118 | 0.733 |  |
|  | (CR5) lack of proper digital competence | 2.93 | 1.083 | -0.055 | -0.329 | 0.658 |  |
| **Computer anxiety (Anxiety)** | (AT1) Working with digital tools makes me nervous. | 2.16 | 1.09 | 0.673 | -0.203 | 0.798 | 0.929 |
|  | (AT2) Digital tools give me an unpleasant feeling. | 2.06 | 1.083 | 0.817 | 0.04 | 0.805 |  |
|  | (AT3) Digital tools make me feel uncomfortable. | 2.19 | 1.172 | 0.654 | -0.4 | 0.730 |  |
|  | (AT4) I feel relaxed when I use digital resources*(R) | 3.78 | 0.857 | -0.255 | -0.578 | 0.179 |  |
| **Perceived usefulness (Usefulness)** | (OR1) Digital tools use can improve my knowledge exchange. | 3.63 | .902 | -.487 | .057 | 0.771 | 0.876 |
|  | (OR2) Digital tools use can enhance self-education. | 3.73 | .904 | -.421 | -.089 | 0.713 |  |
|  | (OR3) Digital tools use would allow me to complete homework more quickly. | 3.78 | .908 | -.190 | -.692 | 0.661 |  |
|  | (OR4) Digital tools use can increase my learning performance. | 3.67 | .925 | -.253 | -.618 | 0.822 |  |
|  | (OR5) Digital tools use can increase my learning efficiency. | 3.69 | .943 | -.480 | -.002 | 0.705 |  |
| **Perceived ease of use (Ease)** | (PEU1) I find digital tools to be easy to use from anywhere. | 3.588 | 0.870221 | -0.27308 | -0.23517 | 0.704 | 0.855 |
|  | (PEU2) Using any digital tools is clear and logical. | 3.472 | 0.83667 | -0.32559 | -0.20274 | 0.628 |  |
|  | (PEU3) Digital tools provide flexibility in interaction with the user | 3.544 | 0.845532 | -0.17928 | 0.024319 | 0.619 |  |
|  | (PEU4) I could easily acquire useful skills needed to use any digital tools | 3.548 | 0.845313 | -0.03116 | -0.3932 | 0.566 |  |
|  | (PEU5) I find digital tools to be easy to use anytime | 3.796 | 0.919656 | -0.52045 | 0.082936 | 0.579 |  |
|  | (PEU6) I can use any digital tools without problems if I have support | 3.42 | 0.995774 | -0.30594 | -0.26122 | 0.606 |  |
|  | (PEU7) I am sure I can use any digital educational resource without technical guidance* |  |  |  |  | 0.523 |  |
|  | (PEU8) I need user instructions for any digital resource* |  |  |  |  | 0.156 |  |
|  | (PEU9) I need help from friends to use any digital resources* |  |  |  |  | 0.094 |  |
| **Behaviour Intention to use (Intention)** | (BU1) Assuming I have permission to use, I will use different digital tools. | 3.732 | 1.027814 | -0.60664 | 0.030408 | 0.649 | 0.809 |
|  | (BU2) I will use different digital tools to search for data, if necessary. | 3.848 | 0.896723 | -0.5043 | 0.192516 | 0.729 |  |
|  | (BU3) I intend to use different digital tools, but after I documented. | 3.472 | 1.010594 | -0.37021 | 0.007869 | 0.696 |  |

*R-Reverse item*; **Eliminated during item analyses The four items eliminated in Stage I were not taken into account in determining the Cronbach's alfa coefficient for each subscale*
