# Supplementary material for: Digital technology adoption scale in the blended learning context in higher education: Development, validation and testing of a specific tool
Source: PLoS One. 2020 Jul 10;15(7):e0235957. doi: 10.1371/journal.pone.0235957 (PMC7351189; doi:10.1371/journal.pone.0235957)
Supplement: S6 Appendix — Convergent and discriminant validity coefficients corresponding to Sample III, N = 262 (a), Sample IV, N = 310 (b) and total sample, N = 572 (c). (DOCX) [file pone.0235957.s006.docx]

**S6 Appendix.** **Convergent and discriminant validity coefficients corresponding to** **Sample III, N=262 (a), Sample IV, N=310 (b) and Total Sample, N=572 (c).**

| \|  \| **CR** \| **AVE** \| **MSV** \| **MaxR(H)** \| **Modern** \| **Ease** \| **Usefulness** \| **Barriers** \| **Anxiety** \| **Traditional** \| **Intention** \| \| --- \| --- \| --- \| --- \| --- \| --- \| --- \| --- \| --- \| --- \| --- \| --- \| \| **Modern** \| 0.894 \| 0.740 \| 0.281 \| 0.938 \| **0.860** \|  \|  \|  \|  \|  \|  \| \| **Ease** \| 0.779 \| 0.639 \| 0.227 \| 0.797 \| 0.184* \| **0.799** \|  \|  \|  \|  \|  \| \| **Usefulness** \| 0.884 \| 0.607 \| 0.606 \| 0.896 \| 0.303*** \| 0.457*** \| **0.779** \|  \|  \|  \|  \| \| **Barriers** \| 0.860 \| 0.552 \| 0.125 \| 0.865 \| 0.098 \| -0.083 \| 0.234** \| **0.743** \|  \|  \|  \| \| **Anxiety** \| 0.941 \| 0.843 \| 0.158 \| 0.950 \| -0.006 \| -0.294*** \| -0.279*** \| 0.354*** \| **0.918** \|  \|  \| \| **Traditional** \| 0.858 \| 0.602 \| 0.341 \| 0.863 \| 0.530*** \| 0.264** \| 0.502*** \| 0.248** \| -0.060 \| **0.776** \|  \| \| **Intention** \| 0.854 \| 0.661 \| 0.606 \| 0.857 \| 0.325*** \| 0.476*** \| 0.779*** \| 0.144* \| -0.398*** \| 0.584*** \| **0.813** \| |
| --- | --- | --- | --- | --- | --- | --- | --- | --- | --- | --- | --- | --- | --- | --- | --- | --- | --- | --- | --- | --- | --- | --- | --- | --- | --- | --- | --- | --- | --- | --- | --- | --- | --- | --- | --- | --- | --- | --- | --- | --- | --- | --- | --- | --- | --- | --- | --- | --- | --- | --- | --- | --- | --- | --- | --- | --- | --- | --- | --- | --- | --- | --- | --- | --- | --- | --- | --- | --- | --- | --- | --- | --- | --- | --- | --- | --- | --- | --- | --- | --- | --- | --- | --- | --- | --- | --- | --- | --- | --- | --- | --- | --- | --- | --- | --- | --- |
| \|  \| **CR** \| **AVE** \| **MSV** \| **MaxR(H)** \| **Modern** \| **Ease** \| **Usefulness** \| **Barriers** \| **Anxiety** \| **Traditional** \| **Intention** \| \| --- \| --- \| --- \| --- \| --- \| --- \| --- \| --- \| --- \| --- \| --- \| --- \| \| **Modern** \| 0.851 \| 0.661 \| 0.411 \| 0.899 \| **0.813** \|  \|  \|  \|  \|  \|  \| \| **Ease** \| 0.853 \| 0.746 \| 0.281 \| 0.910 \| 0.312*** \| **0.864** \|  \|  \|  \|  \|  \| \| **Usefulness** \| 0.886 \| 0.609 \| 0.273 \| 0.886 \| 0.357*** \| 0.408*** \| **0.780** \|  \|  \|  \|  \| \| **Barriers** \| 0.831 \| 0.500 \| 0.172 \| 0.845 \| -0.001 \| 0.066 \| 0.204** \| **0.706** \|  \|  \|  \| \| **Anxiety** \| 0.912 \| 0.777 \| 0.172 \| 0.954 \| -0.072 \| -0.124* \| -0.375*** \| 0.415*** \| **0.882** \|  \|  \| \| **Traditional** \| 0.821 \| 0.535 \| 0.411 \| 0.828 \| 0.641*** \| 0.199** \| 0.411*** \| 0.084 \| -0.061 \| **0.732** \|  \| \| **Intention** \| 0.837 \| 0.632 \| 0.281 \| 0.849 \| 0.377*** \| 0.530*** \| 0.523*** \| 0.031 \| -0.140* \| 0.343*** \| **0.795** \| |
| \|  \| **CR** \| **AVE** \| **MSV** \| **MaxR(H)** \| **Modern** \| **Ease** \| **Usefulness** \| **Barriers** \| **Anxiety** \| **Traditional** \| **Intention** \| \| --- \| --- \| --- \| --- \| --- \| --- \| --- \| --- \| --- \| --- \| --- \| --- \| \| **Modern** \| 0.869 \| 0.693 \| 0.328 \| 0.906 \| **0.832** \|  \|  \|  \|  \|  \|  \| \| **Ease** \| 0.822 \| 0.698 \| 0.255 \| 0.826 \| 0.225*** \| **0.836** \|  \|  \|  \|  \|  \| \| **Usefulness** \| 0.885 \| 0.607 \| 0.420 \| 0.889 \| 0.347*** \| 0.396*** \| **0.779** \|  \|  \|  \|  \| \| **Barriers** \| 0.848 \| 0.528 \| 0.147 \| 0.855 \| 0.061 \| -0.026 \| 0.230*** \| **0.727** \|  \|  \|  \| \| **Anxiety** \| 0.925 \| 0.803 \| 0.147 \| 0.931 \| -0.034 \| -0.196*** \| -0.308*** \| 0.383*** \| **0.896** \|  \|  \| \| **Traditional** \| 0.851 \| 0.590 \| 0.328 \| 0.861 \| 0.573*** \| 0.211*** \| 0.452*** \| 0.160** \| -0.056 \| **0.768** \|  \| \| **Intention** \| 0.844 \| 0.643 \| 0.420 \| 0.844 \| 0.351*** \| 0.505*** \| 0.648*** \| 0.090† \| -0.260*** \| 0.452*** \| **0.802** \| |

*Note: Square roots of AVEs are the diagonal elements highlighted (in bold).*
